# (Reverse) Evolution of a Promiscuous Isochorismate Pyruvate Lyase into an Efficient Chorismate Mutase

**DOI:** 10.1021/acs.biochem.5c00157

**Published:** 2025-07-22

**Authors:** Dominik E. Künzler, Luca Bressan, Linda Jäger, Marianne Gamper, Peter Kast

**Affiliations:** Laboratory of Organic Chemistry, ETH Zurich, CH-8093 Zurich, Switzerland

## Abstract

PchB is an isochorismate pyruvate lyase (IPL) involved in siderophore biosynthesis in . Besides catalyzing the [1,5]-sigmatropic rearrangement of isochorismate, PchB also has weak chorismate mutase (CM) activity, promoting the [3,3]-sigmatropic transformation of chorismate. It has been suggested that the secondary metabolism enzyme PchB evolved from a primary metabolism CM precursor. Here, we employed directed evolution to convert PchB (back) into an efficient CM. A total of seven residues around the active site differing between PchB and a prototypical CM from were randomized, and the resulting gene library was subjected to selection for CM activity. After growth selection in an auxotrophic strain, a catalyst with 10-fold increased CM activity emerged. The improved enzyme was again randomized at three active site positions and subjected to selection, leading to a PchB variant with a *k*_cat_/*K*_m_ of 96,000 M^–1^ s^–1^, which is 40 times higher than that of the parent enzyme and well within the range of dedicated natural CMs. The facile conversion of an IPL into a CM by directed evolution coincides with the fact that both reactions proceed through mechanistically interesting pericyclic processes, reaction types otherwise rarely used by enzymes. When probing typical established CMs for catalytic promiscuity, we discovered spurious IPL activity for the secreted CM from . Our results hint at active site features, particularly a Val at the bottom of the substrate-binding pocket that may have served as a steppingstone for the evolution of IPL activity in a primordial CM.

## Graphical abstract

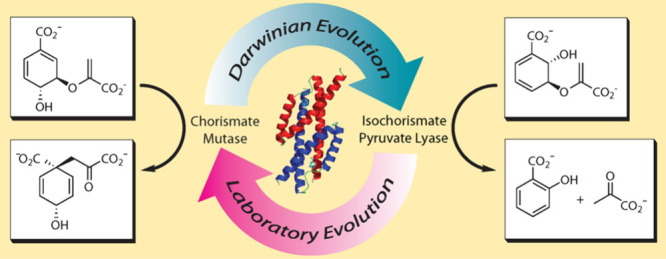

## Introduction

PchB of is a dedicated isochorismate pyruvate lyase (IPL). It catalyzes the elimination reaction of isochorismate to pyruvate and salicylate, which in turn is a precursor of pyochelin and other siderophores used by a few bacterial species to thrive in an iron-limited environmental niche (Figure 1).^1,2^ By using directed evolution,^3^ isotope-labeling, and computation^4,5^ we have established that the IPL reaction is pericyclic. Further structural,^6–8^ mechanistic,^8–10^ and computational^11^ research has supported this finding, making PchB one of only very few enzymes in nature known to catalyze pericyclic processes.^5^ The [1,5]-pericyclic reaction of isochorismate^4,5^ is mechanistically reminiscent^12^ of the [3,3]-sigmatropic (or Claisen) rearrangement of chorismate to prephenate (Figure 1).

**Figure 1:**
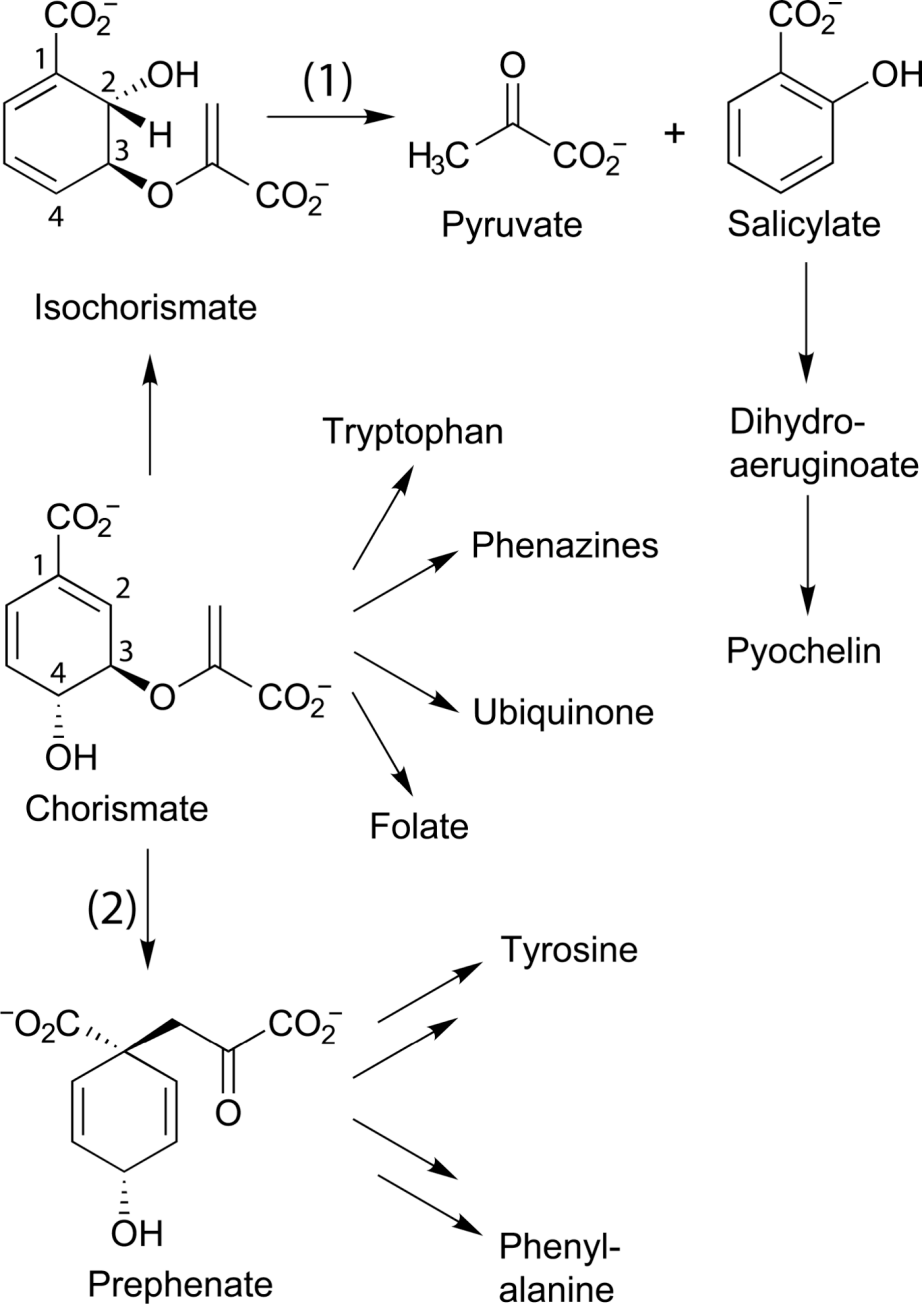
Catalytic tasks of PchB in the metabolism. PchB catalyzes (1) the conversion of isochorismate to salicylate and pyruvate (major IPL activity) and (2) the rearrangement of chorismate to prephenate (additional minor CM activity).

The Claisen rearrangement of chorismate has been the subject of numerous studies showing that, in addition to folding the flexible substrate into a reactive conformation, electrostatic stabilization of the partial charges developing in the transition state is the dominant feature to facilitate this pericyclic reaction.^13–20^ This is illustrated, for instance, by the strategic placement of complementary charged catalytic residues in the active sites of structurally well-studied chorismate mutases (CMs),^19,21–27^ essential enzymes that accelerate the chorismate-to-prephenate rearrangement in the ubiquitous shikimate pathway branching off into the biosynthesis of the proteinogenic aromatic amino acids phenylalanine and tyrosine in bacteria, archaea, fungi, and plants.^28,29^

PchB is encoded in the *pch* operon dedicated to pyochelin siderophore biosynthesis through a rare side branch of the shikimate pathway in .^1^ In many organisms, there exists an alternative, more abundant branch to directly biosynthesize salicylate via isochorismate from chorismate by the evolutionarily unrelated single enzyme salicylate synthase. The latter belongs to the structurally highly homologous MST (menaquinone, siderophore, tryptophan) enzyme group composed of two orthogonal central β-sheets surrounded by α-helices and loops.^30,31^ Whereas the active sites between different MST enzymes are remarkably conserved, there is no match with catalytic features of PchB. In contrast, PchB exhibits low sequence similarity to the ubiquitous CMs of the α-helix-bundle AroQ class,^32,33^ with an overall identity of ca. 20%.^2,3^ An early homology model for the active site of PchB,^2^ which was based on EcCM, a structurally well characterized dimeric CM domain from^23^ and prototype of the α subclass of the AroQ fold family,^34^ was subsequently confirmed by crystal structures of PchB variants with and without ligands.^6,7^ PchB is, like its homologue EcCM, an intertwined dimer of subunits composed of three α-helices each, possessing two identical active sites at the dimer interface.^6^ Several mutagenesis studies have provided information on the function of key active site residues.^2,3,7^

Remarkably, and in addition to its structural relatedness to CMs, PchB also exhibits promiscuous weak CM activity in vitro (CM, *k*_cat_/*K*_m_ = 2 × 10^3^ M^–1^ s^–1^ vs IPL, *k*_cat_/*K*_m_ = 1 × 10^6^ M^–1^ s^–1^)^3^ and complements the phenylalanine and tyrosine auxotrophies of a CM-deficient strain in vivo.^2,3^ Data from PchB model building,^2^ crystal structures,^6,7^ computation,^11^ active site mutagenesis,^2,3,7^ and inhibition kinetics with a transition state analog designed for CMs^3^ indicated that the same active site is used for both the IPL and the CM activities of PchB. They also show that analogous catalytic principles, such as binding of a properly folded conformer of isochorismate and electrostatic stabilization of the transition state, contribute to PchB catalysis. Based on the structural, functional, and mechanistic similarities between PchB, which occurs in a specialized secondary-metabolism pathway,^1^ and the AroQ CMs, ubiquitous in central metabolism,^28,29^ we had previously speculated that IPLs had evolved from an AroQ CM precursor.^2^

If evolution had converted a genuine CM into an IPL, how difficult would it be to develop PchB back into a wild type-like CM? Gaille et al.^2^ attempted to increase the CM activity of PchB by selecting spontaneous mutations in a plasmid-borne wild-type (WT) *pchB* gene harbored by the CM-deficient strain KA12/pKIMP-UAUC^17^ during continuous growth in liquid culture. However, after over 80 generations in minimal medium, a PchB variant with an Ile88Thr mutation emerged that had lost IPL activity while still retaining the same poor CM activity.^2^ Here, we adopted a more focused approach with the goal to convert PchB into an efficient CM by subjecting *pchB* to active site-targeted randomizing mutagenesis coupled with increasingly stringent selection for CM activity.^17,35–37^ The finding that we could readily obtain PchB variants that show dramatically enhanced CM activity suggests that the catalytic machineries in PchB for the chemically distinct pericyclic IPL and CM mechanisms are evolutionarily closely related.

## Experimental Procedures

### Bacterial Strains

For in vivo complementation assays the CM-deficient strain KA12 ^17,38^ was used. Proteins were isolated from the CM-deficient strains KA12, KA13,^33,39^ or KA29.^40^ strain 62-1 transformed with pKS3-02 ^41^ was generously provided by E. Leistner (University of Bonn, Bonn, Germany). The CM-deficient mutator strain KA32-24 (F^–^, λ^–^, *dnaQ*::miniTn*10* (Tet^R^), Δ*(pheA-tyrA-aroF)*, *thi-1*, *endA1*, *hsdR17*, Δ*(argF-lac)­205­(U169)*, *supE44*) is based on strain KB224.^42^ It was constructed by transferring the *dnaQ* (*mutD*) gene mutation (affecting the ε-subunit of DNA polymerase III responsible for the 3′–5′ proofreading exonuclease activity) from strain β1498 (obtained from P. Marlière, University of Évry-Val-d’Essonne, Évry, France) into KB224 by generalized transduction^43^ using the heat-inducible P1 derivative P1Cm112 (obtained from T. Bickle, Biozentrum University of Basel, Basel, Switzerland);^44^ transduced tetracycline-resistant candidates were checked for increased spontaneous mutation to streptomycin resistance and the absence of lysogenic phage.

### Plasmids

Plasmids pKIMP-UAUC,^17^ pKSS-TB,^3^ pMG211,^40^ pKIL10A-W,^3^ pKIL10A-0,^3^ pKTCTET-0,^45^ pET-EcCM-pATCH,^33^ pKTU1-HCW,^40^ and pET-MjCM’-pATCH^33^ were described previously. Plasmids pKTU3-HCT^40^ and pKTU3S-HCT were both used for overproduction of independent batches of *MtCM, the secreted CM from . Plasmid pKTU3S-HCT is identical to pKTU3-HCT,^40^ except that it contains three silent restriction sites (*Asc*I, *Bsm*I, and *Afl*II) in the coding region for *MtCM (M. Gamper, unpublished results). Plasmid pMG211-*MtCMV73E was used to overproduce *MtCM V73E.

To introduce the active site mutation V73E into *MtCM, we conducted site-directed mutagenesis on the gene encoding *MtCM by using pKTU3-HCT as the template for PCR amplification of the 5′ portion of the gene with primers T7fw (5′-TAATACGACTCACTATAGGGGAATT-3′) and *MtCMwoV73Erev (5′-TGTTCTTCTCGGCCGGAATCC-3′) and of the 3′ portion with primers *MtCMwoV73Efw (5′-GGATTCCGGCCGAGAAGAACA-3′) and T7T (5′-TGCTAGTTATTGCTCAGCGG-3′). The two resulting PCR fragments of 216 bp and 506 bp, respectively, were isolated by gel electrophoresis, purified, and subsequently joined by assembly PCR using the outside primers T7fw and T7T. Following restriction digestion by *Nde*I and *Xho*I, the 504 bp fragment containing the gene for *MtCM V73E was ligated to the correspondingly digested 4561 bp fragment of acceptor vector pMG211LJ0 yielding pMG211-*MtCMV73E (5065 bp). To knock out active site arginines for the R49L and R134L variants of *MtCM, site-directed mutagenesis on the template pKTU1-HCW was performed. This was achieved by amplifying the whole plasmid either with the primers *MtCM_R49L_fwd_MS51 (5′-GCTGAGCTGTTGGAGGTCGCCGA-3′) and *MtCM_R49L_rev_MS52 (5′-CTCCAACAGCTCAGCGGCGGCG-3′) for R49L or with *MtCM_R134L_fwd_MS49 (5′-GCATCGCTGTCGGCGATCGACTC-3′) and *MtCM_R134L_rev_MS50 (5′-ATCGCCGACAGCGATGCCGATAGATCC-3′) for R134L. The respective resulting PCR products of 5176 bp or 5178 bp were recircularized using Gibson assembly (New England Biolabs, Ipswich, MA, USA) according to the manufacturer’s instructions. The mutagenized *MtCM genes were amplified with primers LB131_xMtCM-CT_NdeI_fw (5′-ATATCATATGGACGGCACCAGCCAGTTA-3′) and LB132_xMtCM-CT_XhoI_rev (5′-ATATCTCGAGGGCCGGCGGTAGG-3′) resulting in 518 bp fragments. These PCR products were digested with *Nde*I and *Spe*I and the 504 bp fragments ligated to the 5360 bp *Nde*I-*Spe*I fragment of expression vector pET21a (Novagen, Madison, WI, USA) yielding the plasmids pET21a-*MtCMR49L and pET21a-*MtCMR134L (5864 bp).

pMG211LJ0 (5860 bp) was assembled by restriction digestion of pMG211 and pKTCTET-0 with *Nde*I and *Xho*I, and subsequent ligation of the 1299 bp insert fragment from pKTCTET-0 with the 4561 bp vector backbone fragment of pMG211. To introduce the active site mutation V55E into PchB, we conducted site-directed mutagenesis by PCR amplification of wild-type *pchB* carried on pKIL10A-W in two steps. After PCR using the primer pairs T7fw and PchBV55E-NS (5′-ATCGCTGCTTCCCGCTCT-3′) and PchBV55E-S (5′-AGAGCGGGAAGCAGCGAT-3′) and T7T, respectively, the two 274 bp and 238 bp fragments were separated by gel electrophoresis, purified, and subsequently joined by assembly PCR using primers T7fw and T7T. Following restriction digestion, the 344 bp *Nde*I-*Spe*I gene fragment was ligated to the 3944 bp *Nde*I-*Spe*I acceptor fragment of pKIL10A-0 to give pKIL10A-PchBV55E (4288 bp). Plasmid pMG209-AroQ7 was a gift from S. van Sint Fiet (ETH Zurich, Zurich, Switzerland). It was used to overproduce ScCM, the allosterically controlled CM from . The plasmid was constructed by PCR amplification of the *ARO7* gene from genomic DNA from using primers AroQ7S (5′-GGAATTCCATATGGATTTCACAAAACCAGAAACTG-3′) and AroQ7N (5′-CCGCTCGAGCTCTTCCAACCTTCTTAGC-3′), which added the restriction sites *Nde*I and *Xho*I to the 5′ and 3′ end of *ARO7*, respectively. The 772 bp *Nde*I-*Xho*I fragment was ligated with the 5820 bp *Nde*I-*Xho*I fragment from pMG209 ^46^ to give pMG209-AroQ7 (6592 bp). Plasmid pKxPaeCM-HCW (obtained as a gift from K. Würth, ETH Zurich, Zurich, Switzerland) was used to overproduce His-tagged *PaeCM (extracellular CM of ) containing its native N-terminal export sequence. The plasmid contains the wild-type *PaeCM gene amplified from template plasmid pK6041tet-PCM^47^ with primers 768-sPaeCM-W-S (5′- ATCATATGCGCCCGTCGTTCGCC-3′) and 771-sPaeCM-CN (5′- ATCTCGAGCTCGGCCCAGTGACAGAGATC-3′). The PCR product (565 bp) was digested with *Nde*I and *Xho*I and the resulting 555 bp fragment was ligated to the 2797 bp *Nde*I-*Xho*I fragment of pKTCTET-0 to give pKxPaeCM-HCW (3352 bp).

### General DNA Procedures

Plasmid Jetquick spin columns from Genomed (Brunschwig AG, Basel, Switzerland) or DNA purification kits from Zymo Research (Irvine, CA, USA) were used for DNA purification. Other DNA manipulations were according to standard procedures.^48^ Restriction endonucleases and T4 DNA ligase were from New England Biolabs (Ipswich, MA, USA), and HotStarTaq DNA polymerase and the PCR buffer from Qiagen (Basel, Switzerland). Oligonucleotides were custom synthesized by Microsynth (Balgach, Switzerland). DNA sequencing was carried out on an ABI PRISM 310 or an ABI PRISM 3100 Avant Genetic Analyzer using the BigDye Terminator Cycle Sequencing kit (Applied Biosystems, Rotkreuz, Switzerland) or by Microsynth. Fluka/Sigma-Aldrich/Merck (Darmstadt, Germany) was the supplier for all other chemicals. To increase the chances of picking up additional, potentially beneficial mutations, in vitro mutagenesis of isolated library plasmid pools with hydroxylamine was performed according to a published procedure,^49^ except that 8 μg of DNA was used per batch, the NH_2_OH concentration was increased to 1.8 M, and the incubation time was 2 h.

### Construction of the *pchB* Gene Libraries

The mutations for the two gene libraries CM and EVO were introduced by amplification of the truncated *pchB* gene on plasmid pKSS-TB by PCR with HotStarTaq DNA polymerase using pairs of mutagenizing primers, as described previously.^3^ For the CM library, primers 122-LCMS (5′-GTCAGA*gctagc*GAGGCaGCGATTNNKNNKCCaGAGCGGNNKGCaGCGATGCTCCCCGAGCG-3′) and 123-LCMN (5′-GTCAAA*ccgcgg*GTCTGaCGCCAGTACTTGATCTGMNNaGCMNNMNNMNNGTGGATGATCTGCGCGAACAGTC-3′) were used, whereas the EVO library was assembled with primers 189-EVOS (5′-GTCAGA*gctagc*GAGGCAGCGATTTATNKKCCAGAGCGGNNKGCAGCGATGCTCCCCGAGCG-3′) and 188-EVON (5′-GTCAAA*ccgcgg*GTCTGACGCCAGTACTTGATCTGAGCAGCCCTMNNCATGTGGATGATCTGCGCGAACAGTC-3′). Underlined are the randomized codons; equimolar mixtures of bases offered during oligonucleotide synthesis at individual positions are indicated by K (T and G), M (A and C), and N (all four bases); Roman lower case letters indicate silent mutations introduced for optimized gene expression in .^50^ Lower case italics mark, respectively, the restriction sites for *Nhe*I and *Sac*II used to digest the library fragments. The resulting 164 bp fragments were ligated with the 4124 bp *Nhe*I-*Sac*II vector fragment of pKIL10A-0 to yield the 4288 bp library plasmids. Preparative restriction digestion, ligation, and electroporation were carried out as previously stated.^3^ Each library was constructed several times from independently generated PCR fragments, and separately transformed and subjected to selection.

### In Vivo Selection for CM Activity

After electroporation of the library plasmid pools, the transformed cells were washed three times by centrifugation and resuspension in 1× M9 salts,^48^ as described previously.^3^ Selection experiments were carried out in Tyr-free minimal media (M9c/P and M9c/P + F). M9c/P is based on 1× M9 salts and additionally contains 0.2% (w/v) d-(+)-glucose, 1 mM MgSO_4_, 0.1 mM CaCl_2_, 0.1 mM FeSO_4_, 5 μg/mL thiamine-HCl, 5 μg/mL 4-hydroxybenzoic acid, 5 μg/mL 4-aminobenzoic acid, 5 μg/mL 2,3-dihydroxybenzoic acid, 20 μg/mL l-tryptophan, 100 μg/mL sodium ampicillin, 20 μg/mL chloramphenicol, and 100 μM isopropyl-1-thio-β-d-galactopyranoside (IPTG). M9c/P + F, also contains 20 μg/mL l-phenylalanine, leading to faster growth of complementing clones. Before liquid selection, the libraries were characterized by plating aliquots onto M9c/P + FY (M9c/P + F plus 20 μg/mL l-tyrosine) and M9c/P + F minimal medium agar plates (1.5% agar) to determine the library sizes and the fraction of complementing clones.^3^ The diversity of the libraries was calculated as described previously.^51^ For the selection process, 100 μL (10%) of the washed electroporated cells were used to inoculate 30 mL of liquid M9c/P or M9c/P + F medium followed by incubation at 30 or 37 °C at 220 rpm. At appropriate stages, aliquots of dense cultures were plated onto M9c/P ± F minimal plates to obtain isolated colonies for analysis. Single colonies were restreaked onto LB Amp^150^ (150 μg/mL sodium ampicillin) plates, and plasmid DNA from purified clones was isolated and sequenced using primer 04-T7TR.^46^ The ability of each variant to complement the CM deficiency in vivo was confirmed by plasmid retransformation followed by phenotype assessment on minimal plates.

### Protein Production and Purification

Unless stated otherwise, proteins were produced in the CM-deficient strain KA13,^33,39^ which allows for T7 RNA polymerase-driven gene expression. *PaeCM was produced in the CM-deficient strain KA12/pT7POLTS, which provides the T7 RNA polymerase from the helper plasmid pT7POLTS.^45^ Wild-type *MtCM and variants *MtCM V73E, *MtCM R49L, and *MtCM R134L were produced without their periplasmic export sequence in the CM-deficient strain KA29.^40^ Since all proteins possess a His_6_ tag, purification could be performed by nickel-nitrilotriacetic acid (Ni-NTA) affinity chromatography. Following a published protocol^46^ at 30 °C, the above listed production strains, transformed with expression plasmids pKTU3S-HCT, pMG211-*MtCMV73E, pET21a-*MtCMR49L, pET21a-*MtCMR134L, pKxPaeCM-HCW, pMG209-AroQ7, and pKIL10A-W (or its pKIL10A-X gene library derivatives or pKIL10A-PchBV55E), served, respectively, to isolate *MtCM, *MtCM V73E, *MtCM R49L, *MtCM R134L, *PaeCM, ScCM, and PchB (wild type or library variants or PchB V55E; the PchB^H^ format^3^ with an N-terminal Met-His_6_-Ser-Ser-Gly sequence tag was used for all PchB variants). EcCM and MjCM’ (a proteolytically stable C-terminally truncated version of the CM from formerly called )^33^ were overproduced and purified using plasmids pET-EcCM-pATCH and pET-MjCM’-pATCH, respectively, according to published procedures.^33^ The engineered monomeric CM of (mMjCM) was overproduced and purified as published.^33,52^ Protein purity was assessed by sodium dodecyl sulfate polyacrylamide gel electrophoresis using the PhastSystem (GE Healthcare Europe GmbH, Glattbrugg, Switzerland) or the Mini-PROTEAN System (BioRad Laboratories, Hercules, CA, USA). The protein concentration of EcCM, MjCM, mMjCM, ScCM, PchB, and its directed evolutionary descendants were generally determined by UV spectroscopy as described previously.^3^ The concentration of PchB and *MtCM variants investigated for (spurious) CM and IPL activities was determined with the Micro BCA (bicinchoninic acid) assay^53^ with BSA as the concentration standard (Thermo Fisher Scientific, Waltham, MA, USA). Equivalent results within experimental error of the kinetic assays were obtained when using either UV spectroscopy or the Micro BCA assay for the determination of protein concentration.

### Chorismate Mutase and Isochorismate Pyruvate Lyase Activity Assays

Chorismate was produced using strain KA12.^54^ Isochorismate was isolated from strain 62-1 transformed with pKS3-02 ^41^ or from KA12. The steady-state parameters *k*_cat_ and *K*_m_ were derived from the initial rates of substrate disappearance, monitored as an absorbance change at 274 nm (Δε_274_ = 2630 M^–1^ cm^–1^) or 310 nm (Δε_310_ = 370 M^–1^ cm^–1^) for chorismate^55^ and at 278 nm (Δε_278_ = 6640 M^–1^ cm^–1^) for isochorismate.^3^ For each Michaelis–Menten plot, at least 5 independent assays with substrate concentrations typically varying between 5 μM and 2.0 mM for chorismate and between 0.2 μM and typically 100 μM (with 0.5 cm path length cuvettes up to 200 μM) for isochorismate were conducted. Kinetics were measured at 30 °C in 50 mM potassium phosphate buffer (pH 7.5), unless stated otherwise. ScCM activities were assessed in standard buffer additionally containing 10 μM l-tryptophan,^56^ whereas mMjCM was assayed in PBS (10 mM potassium phosphate, 160 mM NaCl, pH 7.5) containing 0.1 mg/mL bovine serum albumin at 20 °C.^52^

A particular emphasis was put on the reliable detection and quantification of spurious IPL activity in established CMs. This required concentrated enzyme solutions with the highest CM concentrations [E] in the activity screens being 44 μM (for EcCM), 9.2 μM (ScCM), 12 μM (mMjCM), 11 μM (MjCM′), and 15 μM (*MtCM, *MtCM V73E, *MtCM R49L, and *MtCM R134L). For measuring potential trace IPL activity (or CM activity for knockout variants) the uncatalyzed substrate decay was determined for each sample. Individual substrate concentrations [S] were first incubated for at least 10 min at 30 °C to record the rate of uncatalyzed substrate conversion (*v*_uncat_). Subsequently, enzyme was added, and the substrate consumption rate was monitored again, allowing for subsequent subtraction of the individual experimental background rate in each sample. Very low IPL activity levels were quantified making use of eq 1 and the Michaelis–Menten equation (eq 2):

$$v_{\mathrm{uncat}}=[S]\cdot k_{\mathrm{uncat}}$$ 1

$$v_{\mathrm{cat}}=(k_{\mathrm{cat}}\cdot [E]\cdot [S])/([S]+K_{m})$$ 2

Assuming that CMs with spurious promiscuous IPL activities have a *K*_m_ for isochorismate above the typically low 10–100 μM substrate concentrations tested, and that a catalyzed rate (*v*_cat_) corresponding to 10% over the background reaction would still be detectable (*v*_cat_ = *v*_uncat_/10), the lowest measurable level of catalytic efficiency can be calculated by eq 3:

$$k_{\mathrm{cat}}/K_{m}=k_{\mathrm{uncat}}/\left(10\cdot \left[E\right]\right)$$ 3

Additional control experiments were performed to verify protein integrity for CMs that lack IPL activity by confirming their published CM activity. Construction and assaying of active site-knockout variants of *MtCM served for excluding nonspecific isochorismate conversion by the mere presence of a high protein concentration. To rule out potential contamination of the isochorismate substrate preparation with chorismate, an isochorismate depletion assay with the sequential addition of two different enzymes was performed at 30 °C. First, isochorismate absorbance at 278 nm was monitored in the presence of a proficient and established CM (*PaeCM, having no IPL activity). Second, the efficient IPL PchB was added to demonstrate that any absorbance decrease at 278 nm beyond the thermal background rate is due to enzymatic isochorismate consumption (Supplementary Figure S1).

### Sequence Alignments and Structural Visualizations

To support decisions of which residues to randomize in the CM and EVO libraries, the Pfam database^57^ (currently hosted on the InterPro Web site of EMBL’s European Bioinformatics Institute; https://www.ebi.ac.uk/interpro/entry/pfam/#table) was consulted. This online resource automatically compiles multiple sequence alignments based on sequence homology of protein domain families. A current search for members of the chorismate mutase protein family of the AroQ class, which also includes the sequence for PchB, returns 53,000 protein sequences grouped as ″Chorismate Mutase Type II domain family″ PF01817 (https://www.ebi.ac.uk/interpro/entry/pfam/PF01817/). At the time of experiment design, it contained, in addition to PchB, 237 sequences that aligned to PchB residues 11–91. Manual counting and subsequent calculation of the residue frequencies at the Pfam precomputed and prealigned^57^ active site positions, as defined from a three-dimensional PchB model,^2^ informed the decision of which positions to randomize in the focused libraries.

Because CMs are ubiquitous in the central metabolism of bacteria, archaea, fungi, and plants, whereas IPLs (belonging to the AroQ class) only occur in a rare secondary metabolic branch of the shikimate pathway in a smaller group of bacterial species, the PF01817 AroQ multiple sequence alignment is composed overwhelmingly of CM rather than IPL sequences. In fact, 92% of the PF01817 sequences were annotated as chorismate mutases whereas only 0.01% (6 out of 53,000) were annotated as IPLs. Among the six is PchB from (accession number Q51507), the only AroQ fold IPL that was biochemically characterized. While it is not possible to distinguish functional CMs and IPLs based on their sequences alone, we aligned all six annotated homologous (presumed) IPL protein sequences in Supplementary Figure S2. A phylogenetic representation of their source organisms, all belonging to the bacterial phylum Pseudomonadota, is shown in Supplementary Figure S3.

For protein sequence alignments, Qiagen CLC Genomics Workbench, Version 23.0.3 (https://digitalinsights.qiagen.com/) was used. Structural superimpositions and graphical representations of three-dimensional structures contained in Protein Data Bank (PDB) files were done with the PyMOL Molecular Graphics System, Version 2.5.2 (https://pymol.org). To create the AlphaFold 3 ^58^ model of the top PchB variant, its protein sequence without the N-terminal His_6_ tag (MMKTPEDCTGLADIREAIDRIDLDIVQALGRRMDYVKAASRFKASEAAIYVPEREAAMLPERARWAEENGLDAPFVEGLFAQIIHMCRAAQIKYWRQTRGAA) was used as input for the online tool (https://alphafoldserver.com/). The option “two amino acid chains” was selected to create the dimer.

## Results

### Targeted Mutagenesis Strategy for Improving CM Activity in PchB

We intended to augment the low CM activity of PchB by mimicking natural evolution through consecutive rounds of mutagenesis of the *pchB* gene coupled to in vivo selection for efficient catalysis. We reasoned that a subset of residues at (and around) the active site that differ between PchB and the prototypical EcCM (or other AroQ class CMs) might be promising mutagenesis targets for evolution of PchB into an efficient CM. Upon visual inspection of a homology model with bound chorismate^2^ and a superimposition of the crystal structures of EcCM^23^ and PchB,^6^ seven amino acid residues of PchB (Pro50, Ala51, Val55, Trp86, Tyr87, Ile88, Glu90) were chosen for mutagenesis in a first gene library (“CM library”). Figure 2 illustrates their location relative to the ligand binding site in PchB and identifies their structural homologues in EcCM.

**Figure 2:**
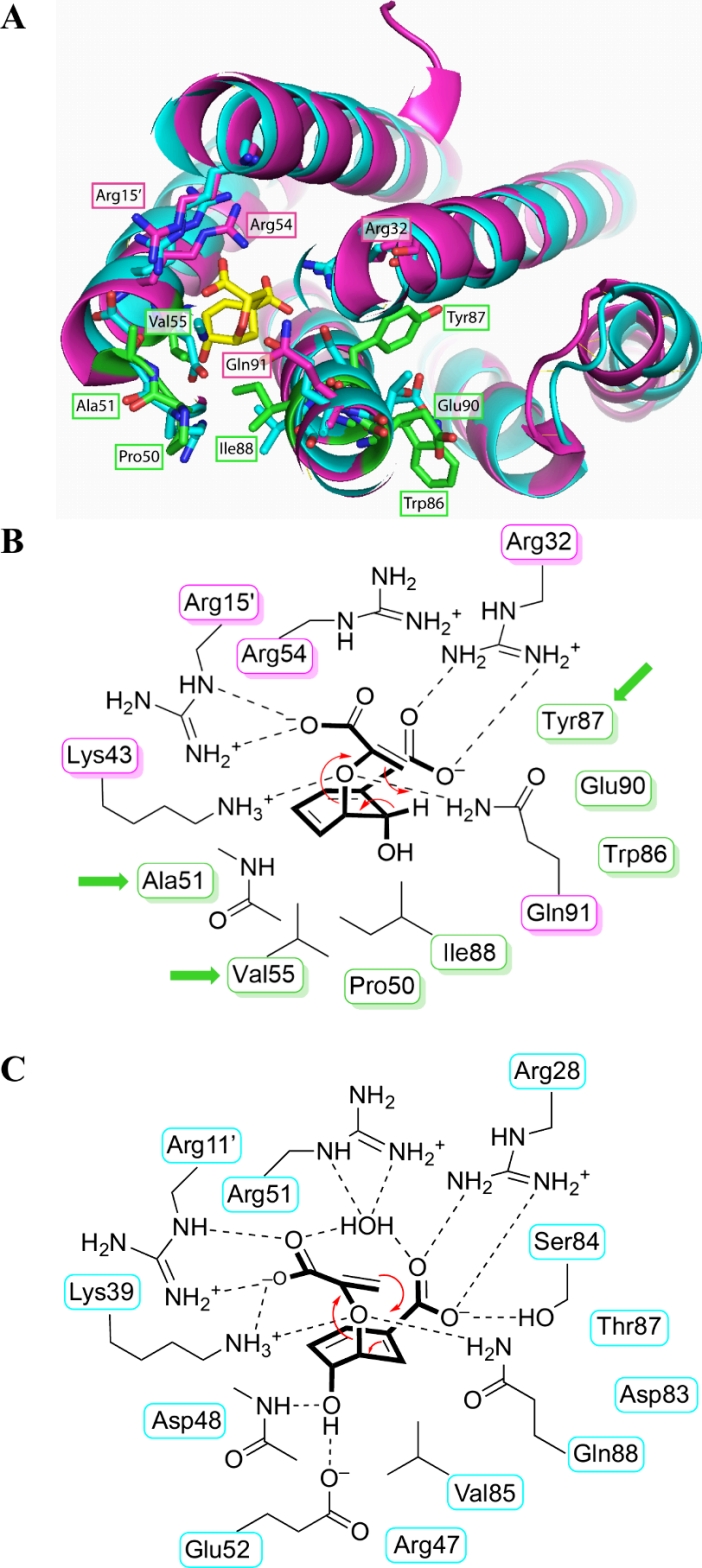
Comparison of the active sites of PchB and EcCM dimers. (A) Superimposition (rmsd 1.115 Å for 153 C_α_ of dimer) of the crystal structures of PchB (backbone in magenta; with closed active site containing a bound pyruvate (omitted); PDB ID: 2H9D)^6^ and EcCM (backbone and residues in cyan; with a bound transition state analog (yellow carbons); PDB ID: 1ECM).^23^ Some catalytic residues were omitted to improve clarity (e.g., the catalytic Lys needed for electrostatic stabilization of developing partial charges in the transition state). PchB residues discussed in this work are shown in magenta (if conserved in EcCM) or in green (if not conserved). (B) Schematic view of a model for the active site of PchB with bound isochorismate (shown with heavy lines) on the basis of PDB ID: 2H9D.^6^ Seven residues (labels framed in green) that are not conserved between PchB and EcCM were targeted for mutagenesis in the “CM library”. The subset with green arrows (positions 51, 55, 87) was randomized in the “EVO library”, which was otherwise based on PchB variant 5-1 (Table 1). (C) Schematic view of a model for the active site of EcCM with bound chorismate (heavy lines), as derived from PDB ID: 1ECM.^23^ Broken lines indicate possible polar interactions between substrate and enzyme. Red reaction arrows specify the [1,5] and [3,3]-sigmatropic rearrangement of isochorismate and chorismate in panels B and C, respectively. The slightly asynchronous initial cleavage of the C–O bond occurring in the pericyclic process results in a partial negative charge on the ether oxygen (not shown here), which is stabilized by active site residues. A prime (′) distinguishes the residues stemming from the second protomer. Note that for consistency with our earlier mutagenesis studies, throughout this work the residue numbering of PchB follows the convention introduced in the initial publications.^2,3^ It corresponds to a +1 increment to the position numbers assigned in the PchB crystal structure in PDB ID: 2H9D.

Inclusion criteria for the randomized CM library residues were (i) having a homologue in EcCM with markedly distinct structural or chemical properties, (ii) being in the immediate vicinity of the active site, and (iii) the PchB residue being less abundant than its EcCM homologue at the corresponding position in an alignment of all 238 AroQ sequences (the “Chorismate Mutase Type II domain family”, PF01817), which were available at the time of library design in the preassembled Pfam Database^57^ (Figure 3). Even though Ile88 of PchB (corresponding to Val85 in EcCM) does not fulfill criteria i and iii, it was still included in the randomized set because it had emerged as a mutational hot spot in an earlier evolution experiment.^2^

**Figure 3:**
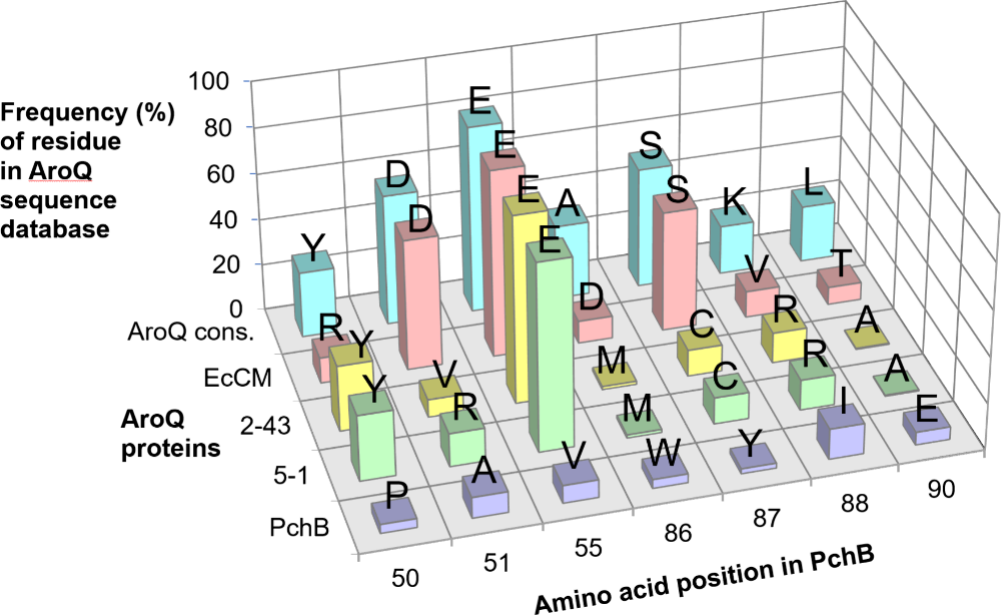
Relative degrees of conservation of residues at the PchB positions relevant for this work within the AroQ class protein family. Included in this analysis were the 238 prealigned AroQ class sequences that made up the “Chorismate Mutase Type II domain family” PF01817 in the Pfam database^57^ at the time of experiment design. This multiple sequence alignment, composed overwhelmingly of CM rather than IPL sequences, covered residues 11–91 of PchB (for more details to this alignment, see the Experimental Procedures section). The database frequencies of the amino acids at each of the seven randomized positions in the CM library are plotted for wild-type PchB, PchB variants 5-1 and 2-43, EcCM, and the AroQ consensus residue. The amino acid identity at the corresponding position is indicated above each column.

### Directed Evolution Scheme and Cycle 1 Selection Results

For construction of the CM Library, PCR fragments of the central portion of *pchB* were generated using long partially randomized oligonucleotide primers, and the library fragment pool was cloned into the selection vector pKIL10A-0 carrying the flanking *pchB* sequences (Figure 4).

**Figure 4:**
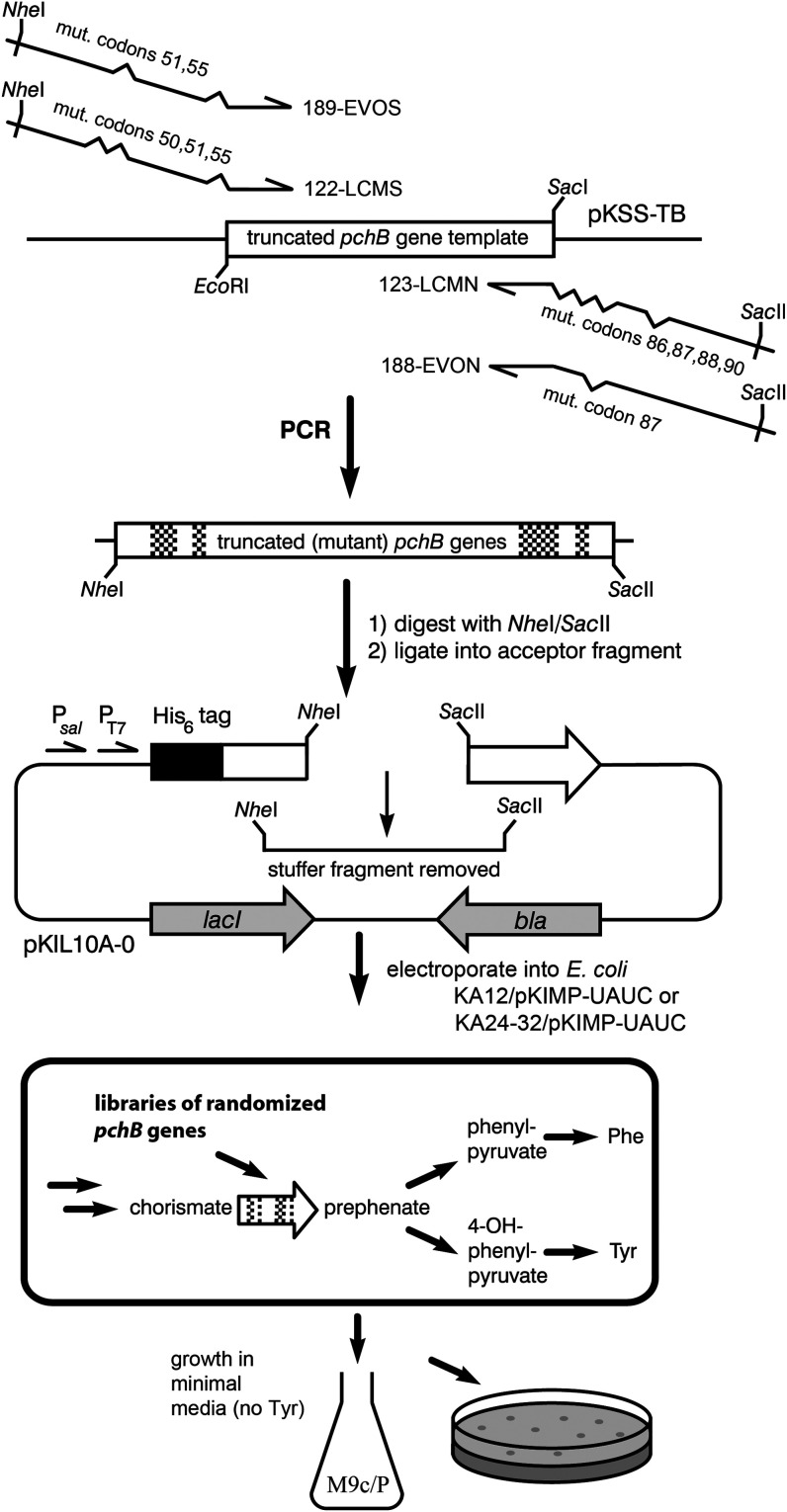
Strategy for gene library construction and genetic selection. The CM and EVO libraries were generated by PCR using a fragment of the *pchB* wild-type gene as template. For the CM library, degenerate primers 122-LCMS and 123-LCMN containing a total of 7 randomized codons for relevant active site-associated residues were incorporated into *pchB*. Primers 189-EVOS and 188-EVON randomize a subset of three of these codons for the EVO library, which is otherwise based on the sequence of the successful variant 5-1 that emerged from the evolutionary Cycle 1. The library fragments were ligated with the vector backbone from pKIL10A-0 obtained after excising a stuffer fragment. CM-deficient host cells were transformed with the ligation products and subjected to growth selection in tyrosine-free M9c/P or M9c/P + F minimal medium, thereby only allowing survival of library members with an efficient CM. Promoters P_*sal*_ and P_T7_ enable *pchB* expression during the growth selection assays and subsequent protein isolation, respectively. *bla* and *lacI* encode, respectively, β-lactamase for ampicillin resistance and the Lac repressor, which controls T7 RNA polymerase-driven gene expression from P_T7_ after transformation of the plasmid into strain KA13.

At the randomized positions, the primer design allowed for codons for all 20 proteinogenic amino acids but only one stop (TAG). Ten independently constructed plasmid library pools were separately electroporated into Phe and Tyr auxotrophic KA12/pKIMP-UAUC cells and subjected to liquid growth selection for CM activity (Figure 5). Control platings indicated that 1.3% of the generated 38,000 variants complemented the CM deficiency of the selection strain.

**Figure 5:**
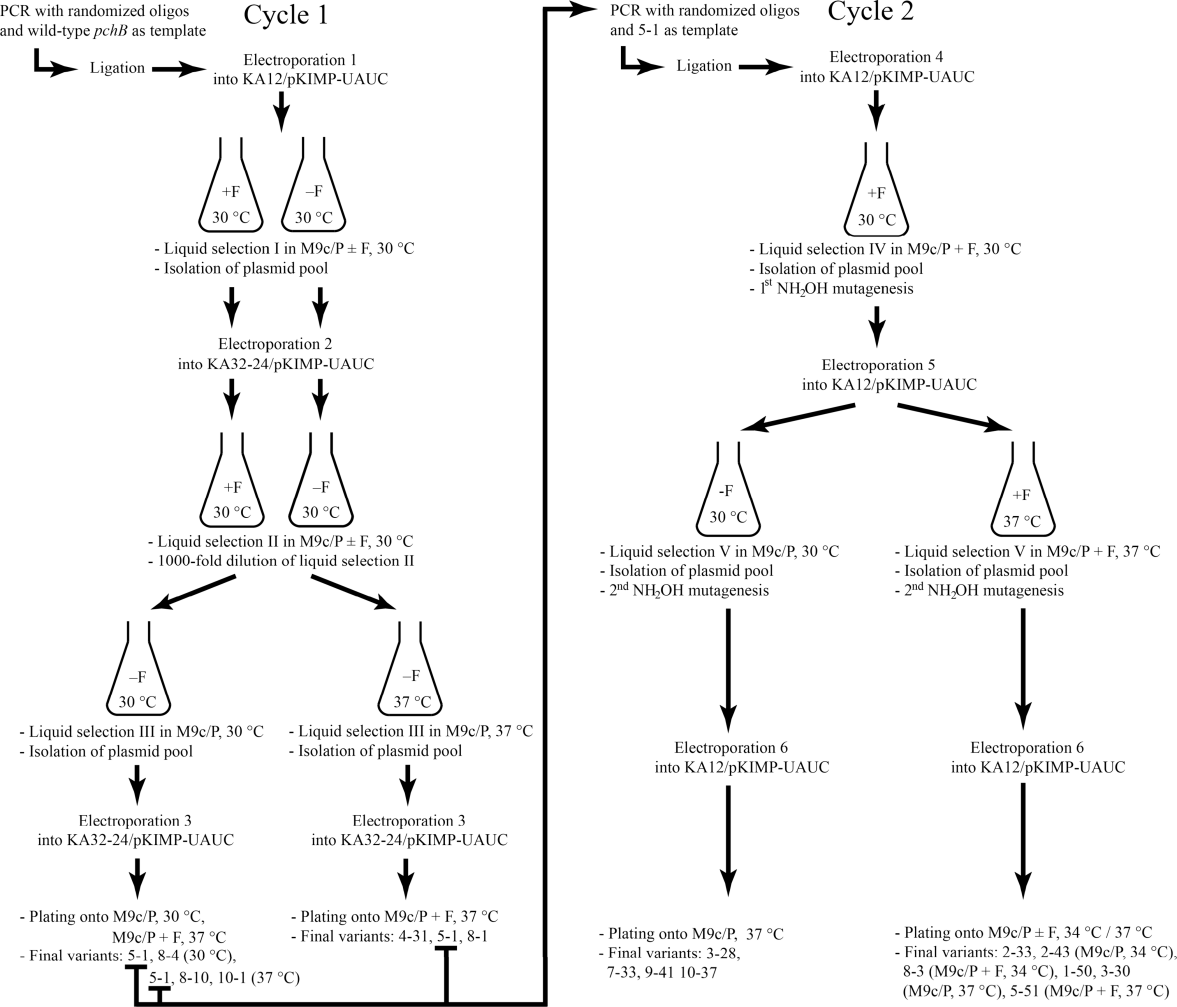
Overview of the directed evolution scheme to improve the CM activity in PchB. In both Cycles 1 and 2, *pchB* was partially randomized by cassette mutagenesis and additionally exposed to mutagenic conditions (mutator strain KA32-24 in Cycle 1 and hydroxylamine treatment of library plasmids in Cycle 2). The selection stringency was varied by altering the incubation temperature and the phenylalanine content in the tyrosine-free M9c/P minimal medium. Complementing clones that were chosen for further characterization are indicated where they emerged during the evolutionary process.

Plasmid pools were isolated from densely grown cultures in selective media followed by electroporation into the CM-deficient mutator strain KA32-24/pKIMP-UAUC, thereby exposing them to an elevated replication error rate in vivo.^59^ The transformants were grown again to high density in M9c/P media at differential selection stringencies. This was followed by a second plasmid pool isolation and reintroduction into fresh KA32-24/pKIMP-UAUC cells, which were subsequently spread onto selective minimal plates (Figure 5). Table 1 shows the amino acids found at the randomized positions in *pchB* genes from six representative independent clones. Even though there was no strict requirement for a particular residue at any of the randomized sites, some trends were already discernible, such as favoring the AroQ CM consensus glutamate at position 55 (instead of the aliphatic WT Val) or a small residue at position 87 (replacing the bulky WT Tyr).

**Table 1:** Amino Acids Found at the Mutagenized Positions in Six Independent PchB Variants after Cycle 1 of Directed Evolution^a^

	residue position^b^						
enzyme variant	50	51	55	86	87	88	90
WT PchB^c^	P	A	V	W	Y	I	E
4-31^d^	R	D	H	A	S	L	R
5-1^d^	Y	R	E	M	C	R	A
8-1^d^	K	S	R	Q	A	E	A
8-4^d^	D	V	S	F	A	V	L
8-10^d^	V	S	E	T	C	T	A
10-1^d^	Y	D	E	A	G	I	S
EcCM^c^	R	D	E	D	S	V	T

a Amino acid residues (one-letter code) were deduced from the corresponding DNA sequences. No additional mutations attributable to possible low-level mutagenesis in the mutator strain were present in the chosen variants.

b For the PchB numbering convention, see legend to Figure 2.

c For comparison, the homologous residues in WT PchB and EcCM are shown.

d The source plate of each listed PchB variant is indicated in Figure 5.

### Randomizing Seven PchB Residues Yields Variants with 10-Fold Increased CM Catalytic Efficiency

Four representative PchB variants obtained after Cycle 1 of directed evolution were purified and their CM activity was determined (Table 2). In all cases, *k*_cat_ for the CM reaction increased at least 2-fold compared to WT PchB. Variant 5-1 showed even a 22-fold increase in its turnover number. The *K*_m_ values of the selected variants tend to be slightly higher than for the WT enzyme—albeit only by a factor of 2 at most (variant 5-1). All four mutants had an increased *k*_cat_/*K*_m_ compared to WT PchB (Table 2) by up to 10-fold (variant 5-1). Thus, the CM activity of WT PchB could already be evolved to a level just 1 order of magnitude lower than that of a dedicated CM, such as EcCM having *k*_cat_/*K*_m_ = 230,000 M^–1^ s^–1^.^55^ At the same time, *k*_cat_/*K*_m_ for the IPL activity of variant 5-1 decreased by 5 orders of magnitude compared to WT PchB (Table 2).

**Table 2:** Catalytic Activities of Evolved PchB Variants in Comparison with WT PchB^a^

	CM activity			IPL activity		
enzyme variant	*k*_cat_	*K*_m_	*k*_cat_/*K*_m_	*k*_cat_	*K*_m_	*k*_cat_/*K*_m_
	(s^–1^)	(μM)	(M^–1^ s^–1^)	(s^–1^)	(μM)	(M^–1^ s^–1^)
WT PchB^b^	0.11	47	2400	1.13	1.2	950,000
Cycle 1 variants						
4-31	0.24	46	5300	NT	NT	NT
5-1	2.4	98	24,000	(0.004)^c^	(400)^c^	13
8-10	0.38	78	4800	NT	NT	NT
10-1	0.45	57	7800	NT	NT	NT
Cycle 2 variants						
2-43	1.7	18	96,000	(0.02)^c^	(200)^c^	110
3-28	0.93	13	71,000	(0.002)^c^	(40)^c^	45
3-30	2.3	280	8200	(0.002)^c^	(300)^c^	7.2
7-33	1.3	38	36,000	(0.003)^c^	(60)^c^	50
10-37	5.9	190	31,000	(0.003)^c^	(100)^c^	26

a Activities were measured in 50 mM potassium phosphate (pH 7.5) at 30 °C; NT, not tested; *k*_cat_/*K*_m_ was calculated from *k*_cat_ and *K*_m_ obtained from data fitting to eq 2 before rounding.^60^ The kinetic CM and IPL data plots for each variant are shown in Supplementary Figures S4–S6, including the correlation coefficient and error of the fit.

b Data from Künzler et al.^3^

c Values in parentheses are rough estimates since the highest isochorismate concentration reliably measurable in this assay is 100 μM (Supplementary Figure S6).

### Library Design and Selected Sequence Patterns after Second Round of Directed Evolution

To boost the CM activity even further, another evolution experiment (Cycle 2) was designed based on the amino acid sequence of the most active variant discovered in Cycle 1. For the “EVO library”, the codons for positions 51, 55, and 87 were randomized within the sequence context of clone 5-1. Residues corresponding to WT PchB Ala51 and Val55 are conserved in the strongly CM-dominated AroQ class alignment primarily as Asp and Glu (with frequencies of 57 and 81%, respectively; Figure 3), whereas sequences with Ala and Val, respectively, are much less abundant. Positions 51 and 55 were thus considered promising candidates for randomization in the EVO library to evolve a better CM. As illustrated in Figure 2C, the EcCM residue Asp48 corresponding to Ala51 of PchB interacts with the 4-hydroxyl group of the ligand through its backbone amide;^23^ it is conceivable that the structure of the side chain influences the precise positioning of the H-bonding peptide unit. The residue at position 55 is thought to contribute to packing with the isochorismate ring (Figure 2B).^2^ It thus appeared plausible that mutation to a residue capable of favorable interactions with the 4-hydroxyl group of chorismate, in analogy to Glu52 in EcCM (Figure 2C), could result in increased CM activity of corresponding PchB variants. Position 87 is, after positions 55 and 51, the third most conserved CM library position in our AroQ sequence comparison (Figure 3). Moreover, the tyrosine present in PchB at this site is very rare in AroQ family members (seen in only 2% of the sequences, Figure 3), making it a candidate for an IPL-specific feature. Thus, mutating position 87 was deemed promising for improving CM activity.

Using the strategy outlined in Figure 4, eight independently assembled EVO gene libraries were transformed into KA12/pKIMP-UAUC cells. The initial combined library size was 35,600 clones, statistically covering 66% of all theoretically possible *pchB* genes and 99% of all possible PchB protein variations for the three randomized positions. 8.5% of these clones complemented the CM deficiency. The eight individually transformed libraries were each subjected to two evolutionary rounds consisting of growth selection in separate minimal medium cultures followed by plasmid pool isolation, exposure to the low-level mutagen hydroxylamine, and retransformation, as illustrated in Figure 5.

After the final retransformation, plasmids from ten randomly selected colonies growing on selective M9c/P ± F minimal plates were isolated and sequenced. Table 3 lists the amino acids selected at the randomized positions in the encoded PchB variants.

**Table 3:** Amino Acids Found at the Mutagenized Positions in Ten Independent PchB Variants after Cycle 2 of Directed Evolution^a^

	residue position^b^		
enzyme variant	51	55	87
WT PchB^c^	A	V	Y
1-50^d^	I	E	G
2-33^d^	N	E	L
2-43^d^	V	E	C
3-28^d^	Q	E	C
3-30^d^	R	E	A
5-51^d^	A	L	M
7-33^d^	Q	E	Y
8-3^d^	R	E	C
9-41^d^	S	E	A
10-37^d^	R	E	S
EcCM^c^	D	E	S
5-1^c^	R	E	C

a Amino acid residues at the randomized positions of PchB variants from the EVO library as deduced from the corresponding DNA sequences. No additional mutations attributable to the hydroxylamine treatment of the isolated plasmid DNA pool in between the growth selection steps had emerged in these variants. The protein sequences of the listed PchB variants additionally contain the four 5-1 substitutions P50Y, W86M, I88R, and E90A relative to WT PchB.

b For the PchB numbering convention, see legend to Figure 2.

c For comparison, the residues at the corresponding positions in WT PchB, EcCM, and the top variant emerging from Cycle 1 (5-1) are shown.

d The source plates of individual PchB variants are indicated in Figure 5.

The results confirmed the trends already observed in Cycle 1 (Table 1) to favor a larger/more polar replacement at position 51 and a small/polar residue at position 87 (Cys, Ser, Ala, Gly). The slight preference for glutamic acid at position 55 seen in Cycle 1 was impressively consolidated with 90% of the variants now exhibiting Glu55. Five (clones 2-43, 3-28, 3-30, 7-33, 10-37) of the 10 sequenced variants from Cycle 2 were chosen for in-depth characterization. For all clones, their ability to complement the CM deficiency upon retransformation of the corresponding plasmids into the selection strain was confirmed, both at 34 and 37 °C.

### Highly Efficient CM Catalysts Found after Just Two Evolution Cycles

The five PchB variants were purified and their catalytic parameters determined (Table 2). The *k*_cat_ values for the CM activity varied between 0.93 and 5.9 s^–1^, corresponding to a range of 0.4 to 2.5-fold the *k*_cat_ of the parental variant 5-1. The *K*_m_ values of three of the five variants were more than 2-fold improved compared to 5-1 and even were lower than that of WT PchB. The catalytic efficiencies *k*_cat_/*K*_m_ of four of the five analyzed variants increased relative to 5-1 by 1.3 to 4-fold. The top variant 2-43 showed a *k*_cat_/*K*_m_ that was 40 times better than WT PchB. The five variants were also characterized with respect to their IPL activity (Table 2). The *k*_cat_/*K*_m_ values varied between half and 4 times the value for the starting enzyme 5-1, which had already suffered a loss of 5 orders of magnitude in catalytic IPL efficiency relative to WT PchB.

### Vice Versa: Assessing Genuine AroQ CMs Identifies Secondary IPL Activity in *MtCM

Since WT PchB naturally shows weak promiscuous CM activity, which was shown here to be readily augmentable by directed evolution to levels approaching that of natural CMs, we wondered whether there exist established dedicated CMs that exhibit weak promiscuous IPL activity. Several well-characterized AroQ class CMs were therefore purified and checked for exhibiting IPL catalysis in addition to their previously known efficient CM activity. None of the tested CMs showed convincing IPL activity over the background rate except for the extracellular CM of (*MtCM),^40^ which does indeed turn over isochorismate as an alternative substrate, albeit with a 2.4 × 10^5^ times lower *k*_cat_/*K*_m_ than PchB (Table 4 and Supplementary Figure S7A).

**Table 4:** Catalytic Activities by Wild-Type PchB, Established CMs, and Engineered Variants^a^

	CM activity			IPL activity		
AroQ enzyme	*k*_cat_ (s^–1^)	*K*_m_ (μM)	*k*_cat_/*K*_m_ (M^–1^ s^–1^)	*k*_cat_ (s^–1^)	*K*_m_ (μM)	*k*_cat_/*K*_m_ (M^–1^ s^–1^)
PchB	0.096 ± 0.028	41 ± 5	2300 ± 600	1.02 ± 0.11	1.01 ± 0.24	1,040,000 ± 160,000
PchB V55E	0.23 ± 0.01	350 ± 30	680 ± 30	0.67 ± 0.13	11.1 ± 0.5	60,000 ± 9000
*MtCM	49 ± 11	170 ± 16	300,000 ± 40,000	(0.0009 ± 0.0002)^b^	(200 ± 30)^b^	4.4 ± 0.4
*MtCM V73E	13.6 ± 2.8	560 ± 60	24,000 ± 2000	NA^c^	NA^c^	0.67 ± 0.03^c^
*MtCM R49L	0.0070 ± 0.0019	220 ± 80	33 ± 4	NA^d^	NA^d^	<0.15^d^
*MtCM R134L	ND^b^	ND^b^	17 ± 4	NA^d^	NA^d^	<0.15^d^
EcCM	55/*69* ^e^	240/*300* ^e^	230,000	NA^d^	NA^d^	<0.5^d^
MjCM′	5.5/*5.7* ^e^	16/*41* ^e^	340,000	NA^d^	NA^d^	<0.2^d^
mMjCM	3.5/*3.2* ^e^	270/*170* ^e^	13,000	NA^d^	NA^d^	<0.2^d^
ScCM	37/*13* ^e^	590/*600* ^e^	62,000	NA^d^	NA^d^	<1^d^

a All enzymes were assayed in 50 mM potassium phosphate buffer (pH 7.5) at 30 °C, except for mMjCM (10 mM sodium phosphate, pH 7.5, 160 mM NaCl, 0.1 mg/mL bovine serum albumin; at 20 °C) and ScCM (50 mM potassium phosphate, pH 7.5, 10 μM l-tryptophan; 30 °C). NA, not applicable; *k*_cat_ is calculated per active site; the reported *k*_cat_/*K*_m_ ratios represent the mean (and standard deviation σ_n‑1_) from averaging *k*_cat_/*K*_m_ values derived directly from the *k*_cat_ and *K*_m_ values obtained from separate iterative fitting of the data of at least two true biological replicates to the Michaelis–Menten equation (eq 2).^60^ The mean and standard deviations for wild-type PchB kinetics stem from six independent biological replicates. Enzyme concentrations for PchB V55E and *MtCM variants were determined by the BCA assay. For further details, see the Experimental Procedures section.

b Here, individual *k*_cat_ and *K*_m_ values are either rough estimates (if in parentheses) or not determinable (ND) because the highest technically measurable chorismate or isochorismate concentrations in the assay were either too close to or below the *K*_m_ of the corresponding variant.

c The lack of discernible substrate saturation even at 200 μM isochorismate, the highest practical substrate concentration accessible in these assays, precluded estimation of individual *k*_cat_ and *K*_m_ values, whereas the calculation of the genuine second-order rate constant *k*_cat_/*K*_m_ is still reliable under these subsaturating conditions.

d Since no substrate saturation was perceived in the kinetic traces between 10 and 200 μM isochorismate, the lowest practical detection level for any possible catalytic activity above background was quantified according to *k*_cat_/*K*_m_ = *k*_uncat_/(10·[E]) (see the Experimental Procedures section for details of the calculation).

e Independent literature values are provided in italics for comparison for EcCM,^55^ MjCM′,^33^ mMjCM,^52^ and ScCM.^56^

The IPL reaction catalyzed by *MtCM shows Michaelis–Menten-type substrate saturation kinetics with a tentative *K*_m_ around 200 μM (Supplementary Figure S8). *MtCM is a representative of the AroQ_γ_ subclass, encompassing exported CMs with a topologically rearranged AroQ fold.^25,40^ Sequence alignments with PchB, EcCM, and *PaeCM (AroQ_γ_ subclass CM from ) show that *MtCM features a valine residue (Val73) at the position homologous to Val55 in PchB (Figure 6A) instead of the glutamic acid clearly prevailing in AroQ CMs (Figure 3). Note that Glu is the predominant residue in the other AroQ_γ_ subclass CMs,^61^ including in *PaeCM (Figure 6A).

**Figure 6:**
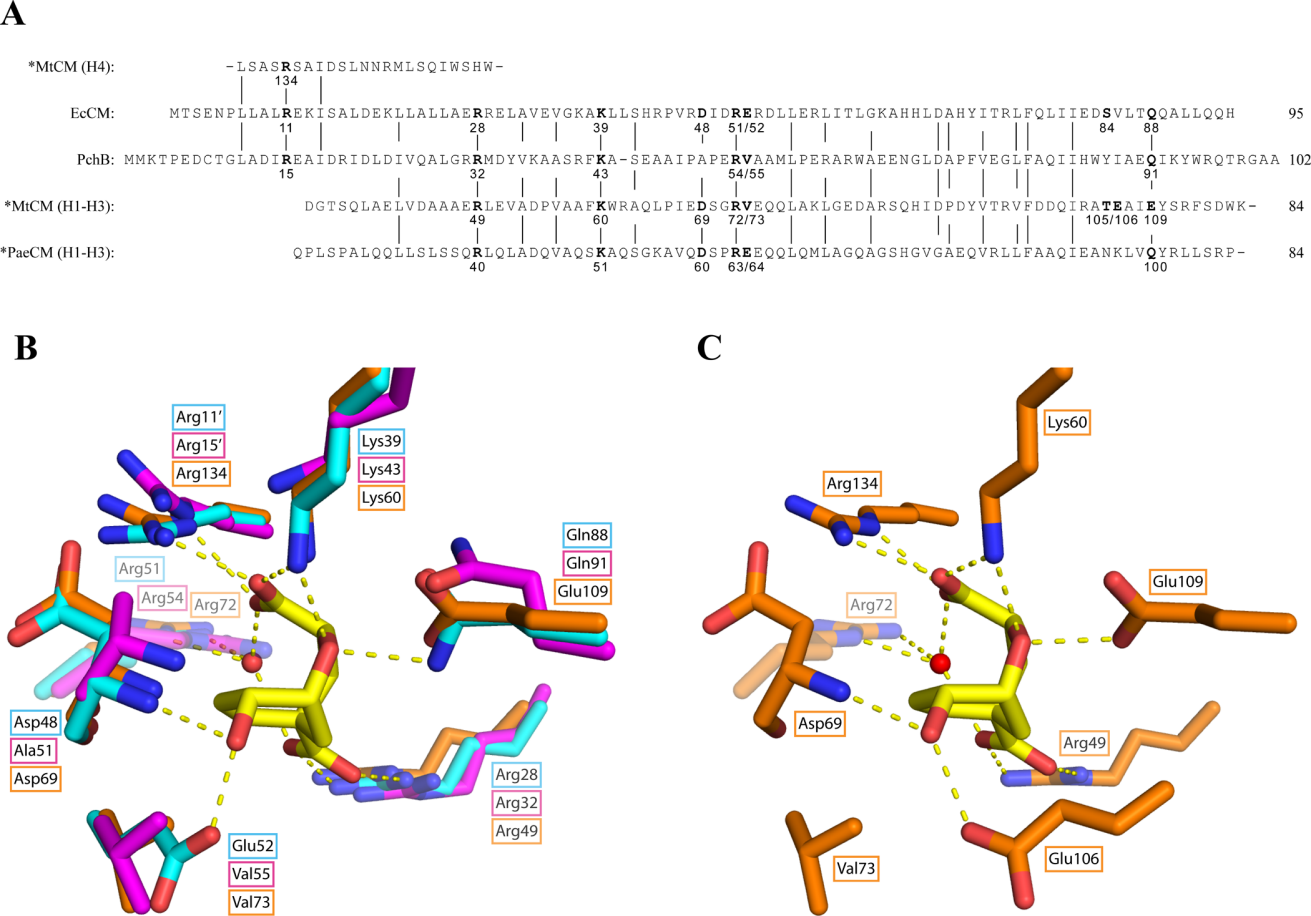
Sequence and structural homology of critical AroQ active site features. (A) Sequence alignment of PchB and EcCM and the homologous segments from the AroQ_γ_ subclass *MtCM and *PaeCM, in analogy to the structure-based alignment published previously.^25^ Important active site residues are numbered and shown in bold. Vertical lines relate homologous residues that are identical in at least three of the four aligned proteins. Note that AroQ_γ_ subclass members possess a rearranged helix topology, resulting in noncontiguous numbering of structurally and functionally homologous stretches, such as helix 4 (H4) of *MtCM. (B) Superimposition of selected active site residues from the crystal structures of PchB (magenta; PDB ID: 2H9D),^6^ EcCM (cyan; with a bound transition state analog (yellow carbons); PDB ID: 1ECM),^23^ and *MtCM (orange; PDB ID: 2FP2).^25^ For clarity, only the side chain residues are depicted, except for the amide backbone of EcCM Asp48 (and of PchB Ala51 and *MtCM Asp69), which also interacts with the transition state analog. Dashed yellow lines represent polar interactions between EcCM residues and the bound transition state analog. (C) Illustration of *MtCM active site residue interactions with the bound transition state analog in the crystal structure of the *MtCM-ligand complex (PDB ID: 2FP2),^25^ as described in panel B. Interestingly, Glu106 serves as a substitute for Val73 to engage in H-bonding with the ligand’s OH-group. Note that for the secreted CMs (*MtCM and *PaeCM), residue numbering starts with the initial Met of the reading frame, i.e., it includes the leader peptide, consistent with the convention introduced in the initial papers.^25,40^

Because the observed IPL activity of *MtCM was only minimal, we performed a series of control experiments to rule out substrate contaminations or nonspecific catalysis due to the necessary presence of very high protein concentrations in the assay to detect any activity. These experiments included exclusion of contamination by traces of chorismate in the used isochorismate substrate batch. This was accomplished by showing that incubation with *PaeCM, a representative dedicated and highly active CM, did not decrease the isochorismate absorption beyond the thermal background reaction (Supplementary Figure S1). Furthermore, we constructed two *MtCM variants carrying point mutations in active site Arg residues (either R49L or R134L, Figure 6), thereby cutting down *k*_cat_/*K*_m_ for CM activity by 4 orders of magnitude (Table 4). IPL assays with variants *MtCM R49L and *MtCM R134L demonstrated that the mere presence of 15 μM protein does not by itself result in isochorismate decay rates above background (Table 4 and Supplementary Figure S7B).

### Isolated Val to Glu Interchange Impairs Both IPL and CM Activities of PchB and *MtCM

Our directed-evolution results suggest that *k*_cat_/*K*_m_ values ≥10^4^–10^5^ M^–1^ s^–1^ for the CM reaction require the emerging Glu55 in PchB variants; however, this also coincides with a 10^4^–10^5^-fold reduction in IPL activity. Conversely, a valine at the homologous position in AroQ enzymes correlates with IPL activity, as found for the natural CM *MtCM, which has Val73 at the corresponding site (Figure 6). To explore whether one single amino acid exchange within a given natural AroQ active site configuration is sufficient to shift the reaction scope of the AroQ enzyme, we constructed rational point mutants of WT *MtCM and PchB. After replacing the respective wild-type Val73 and Val55 by the AroQ CM consensus residue Glu we determined remaining IPL and CM activities of the engineered variants. Indeed, the IPL activity of *MtCM V73E dropped by an order of magnitude to barely above background (*k*_cat_/*K*_m_ = 0.67 M^–1^ s^–1^; Table 4 and Supplementary Figure S7A). However, this variant also suffered a 13-fold reduction in CM activity, suggesting that the change of the aliphatic Val73 to the charged Glu side chain alone is clearly detrimental for an active site already optimized for high CM catalysis. Kinetic investigation of the PchB V55E variant revealed that an isolated V55E replacement caused a 17-fold reduced *k*_cat_/*K*_m_ for the IPL activity, but also a 3-fold overall decrease in the catalytic efficiency of its promiscuous CM function (Table 4), again pointing to a significantly perturbed active site structure. Notably, the loss in *k*_cat_/*K*_m_ is predominantly due to an increase in *K*_m_ by 1 order of magnitude for both inherent PchB activities, suggesting interference with substrate binding as a result of the Val-to-Glu exchange. In contrast, the rate constant *k*_cat_ for CM catalysis was even improved 2-fold in PchB V55E compared to the WT enzyme.

## Discussion

### Reverse Evolution of PchB into a Highly Active Chorismate Mutase

By applying two consecutive cycles of directed evolution, it was possible to boost *k*_cat_/*K*_m_ for the low promiscuous CM activity of WT PchB by a factor of 40. This resulted in a CM activity for the top variant 2-43 just two to 3-fold below that of EcCM, a genuine CM (Tables 2 and 4). The evolution of a catalyst for a [3,3]-sigmatropic Claisen rearrangement from an enzyme catalyzing a [1,5]-pericyclic reaction was remarkably efficient. Already the first evolutionary cycle yielded an increase in CM activity by 1 order of magnitude. In Cycle 2, an improvement in *k*_cat_/*K*_m_ by another factor of 4 was attained. Since WT PchB intrinsically possesses modest CM activity, we had speculated previously that the efficient IPL catalysis provided by today’s *pchB* gene product in a rare side branch of the shikimate pathway (Figure 1)^1^ might have evolved after gene duplication of an ancient *aroQ* gene encoding a dedicated ubiquitous AroQ class CM, central for aromatic amino acid biosynthesis.^2^ This builds on the notion already discussed more than five decades ago that new activity can arise by evolutionary pressure on a duplicated copy of an enzyme-encoding gene.^62^ Upon analyzing the rates of diversification before and after fixation of the new function^63^ and by considering more recent models and experiments, this hypothesis was later extended to suggest that diverging gene products already showed promiscuity or dual-function prior to gene duplication.^64–68^ The discovery of many contemporary enzymes that possess secondary, additional, or “moonlighting” activities support this idea.^69–72^ A bifunctional ancestor of PchB that emerged in such a process might conceivably have been subjected to evolutionary pressure for higher IPL activity useful for a specialized secondary metabolic pathway, such as the one described for siderophore biosynthesis in^1^ and possibly in other species from the phylum Pseudomonadota (Supplementary Figure S3).

The promiscuous PchB could be considered in such a scenario as a highly evolved intermediate in the evolutionary trajectory from a dedicated CM to a yet to be discovered fully specialized IPL, and thus represent a “snapshot” of an evolving enzyme. By taking advantage of the residual promiscuous CM activity, we have converted PchB into an efficient CM by reversing the presumed evolutionary trajectory. This process may have worked particularly well in our case because most of the important residues involved in the catalytic machinery of AroQ CMs are already in place in WT PchB. In fact, ten out of the 19 EcCM residues within a 6 Å sphere around the bound transition state analog in Figure 2A are fully conserved in PchB.^2^ Also, the substrates of PchB—isochorismate and chorismate—are structurally similar, differing only in the position of the hydroxyl group (2-OH in isochorismate and 4-OH in chorismate; Figure 1). The hydrophobic side chain of PchB’s Val55, one of the few deviating residues at the bottom of the active site in Figure 2, seems perfectly positioned for hydrophobic interactions with C4 of isochorismate. An alignment of the just six annotated bona fide IPLs among all 53,000 preassembled AroQ sequences in the “Chorismate Mutase Type II domain family” PF01817 of the Pfam database,^57^ reveals that Val55 of PchB is conserved in four of the six sequences (Supplementary Figure S2). The other two sequences have Phe and Tyr at this site, potentially also allowing for hydrophobic interactions with isochorismate. While the consensus of all seven randomized positions generally coincides with the identity of the PchB residue, it should be noted, however, that besides PchB, IPL activity was not confirmed for any of the five other aligned proteins.

A Glu residue able to interact with chorismate’s hydroxyl group is highly conserved in the family of AroQ proteins at the position corresponding to Val55 in PchB, according to the multiple sequence alignment PF01817, which is dominated by the ubiquitous AroQ CMs (Figure 3).^57^ In the EcCM crystal structure, the side chain carboxylate of the corresponding Glu52 residue was shown to interact with the 4-OH group of chorismate,^23^ whereas such an interaction is not possible with Val55 of WT PchB (Figures 2 and 6B). In fact, also in other structurally characterized CMs, including the AroQ proteins ScCM,^24,73,74^ *MtCM (Figure 6C),^25^ MtCM,^27^ and CgCM^75^ and even in the evolutionarily unrelated AroH class BsCM,^21,22^ a glutamate residue is prominently positioned for interaction with the ligand’s hydroxyl group. Thus, selection of Glu at position 55, as found in most of the successful variants including 5-1 and 2-43 (Table 3), is a coherent result given that our evolution experiment aimed at converting PchB into an effective CM.

### Key Residues for High CM Activity

The importance of the emerging Glu55 during directed evolution (Table 3) is also supported by the results of previous (combinatorial) mutagenesis experiments with both AroQ and AroH CMs,^76–78^ suggesting that its interaction with the 4-OH group of chorismate is critical for efficient CM activity. Despite this insight, switching between CM and IPL catalysis could not easily be accomplished by simply exchanging the corresponding Val residue by Glu in the native enzymes *MtCM and PchB. In fact, just swapping Val73 of *MtCM for the AroQ consensus glutamate affected both CM and IPL catalysis by lowering *k*_cat_/*K*_m_ for each activity by an order of magnitude (Table 4). Moreover, while the single Val55-to-Glu55 exchange in PchB decreased the *k*_cat_/*K*_m_ for IPL activity by 17-fold, the efficiency of its promiscuous CM catalysis also dropped by 3-fold. Still, retaining the valine appears more important for the native IPL function in the WT PchB background than for its promiscuous weak CM activity. In fact, changing to a glutamate at position 55 even augmented *k*_cat_ of the CM reaction by a factor of 2 (Table 4). This is consistent with the kinetic parameters for variants evolved in this study (Table 2) and with the importance of an acidic side chain at this location found in previous combinatorial mutagenesis experiments.^76–78^ Overall, however, replacing the aliphatic nonpolar valine with the polar glutamate at the bottom of the substrate binding site may interfere with the proper alignment of active site residues. Indeed, both rationally designed variants *MtCM V73E and PchB V55E suffer from impaired substrate binding, as reflected by the strongly increased *K*_m_ values for chorismate and isochorismate. Obviously, these isolated substitutions in the active site are detrimental when on their own, highlighting the importance of epistatic effects, i.e., the critical need for accompanying enabling mutations to permit successful evolutionary trajectories.^79–81^

PchB variant 5-1, which has the highest CM activity after one round of directed evolution, contains the AroQ consensus residues Glu and Tyr at positions 55 and 50, respectively (Figure 3). Whereas its Arg51 corresponds to the second-most frequent residue in AroQ proteins, the 4-fold increase in CM activity between the most active Cycle 1 variant (5-1) and the top Cycle 2 variant (2-43) is solely attributable to the R51V exchange. The bulky Arg51 of variant 5-1 may be less favorable for CM activity than the smaller valine in variant 2-43 (modeled in Figure 7A) due to interference with optimal binding of the 4-hydroxyl group of chorismate. In fact, EcCM Asp48 (homologous to PchB position 51) hydrogen-bonds via its peptide backbone amide with the hydroxyl group of the transition state analog of the CM reaction (Figures 2 and 6).^23^ In addition, Arg51 of variant 5-1 may unfavorably interact with the newly emerging Glu55, as it is likely that residues at positions 51 and 55 act in concert at the active site. They must maintain a balance between facilitating the formation of the conducive transition state, e.g., by Glu55 stabilizing developing positive charge on the cyclohexadienyl ring mediated through the 4-OH group, and at the same time allow for good packing and hydrogen bonding to the ligand (by Val51 and Glu55; Figure 7B).

**Figure 7:**
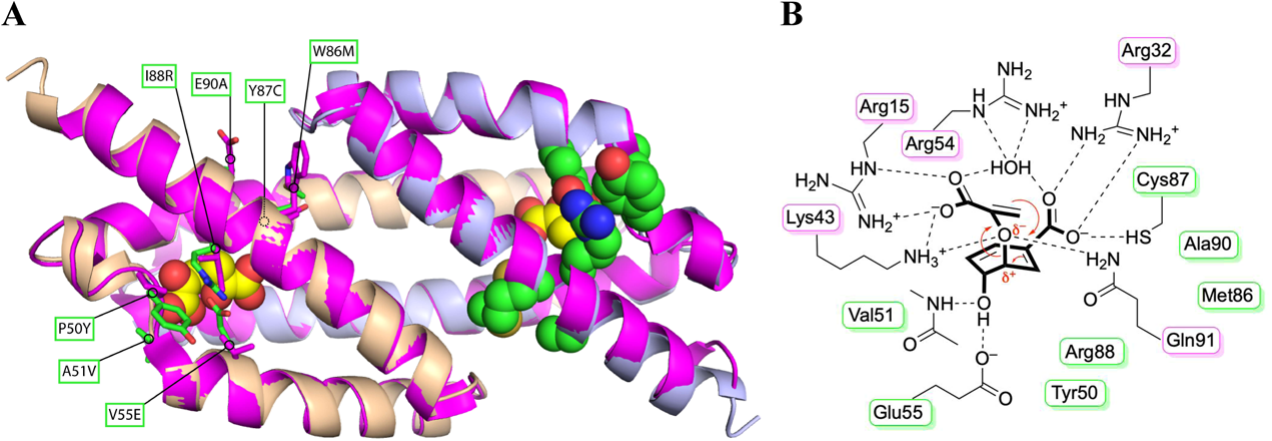
AlphaFold 3 model of the selected winner variant 2-43. (A) Structure of the top-evolved enzyme 2-43 modeled using AlphaFold 3 ^58^ upon providing its primary sequence as input. The two protomers are depicted in wheat and light blue. The side chains of the residues randomized to evolve 2-43 are highlighted in the AlphaFold 3 model with green carbons as sticks (active site on the left) or as spheres (right). The 2-43 model is superimposed onto the crystal structure of WT PchB (magenta; PDB ID: 2H9D),^6^ including its cocrystallized ligand pyruvate (yellow spheres) to indicate the location of the active sites. Labels pinpoint the amino acid replacements from WT PchB (magenta sticks) to the evolved 2-43 (green sticks). (B) Schematic representation of the active site of PchB variant 2-43 showing hypothetical interactions with a chorismate molecule that undergoes a slightly asynchronous [3,3]-sigmatropic Claisen rearrangement (red arrows; partial charges upon initiating C–O bond cleavage shown). The residues of variant 2-43 selected from the randomized libraries are highlighted with green frames, whereas magenta frames indicate active site residues that were kept constant during evolution and still correspond to those of WT PchB.

This intricate balance of steric fit and electrostatics, particularly at the bottom of the active site and beyond, is what makes the promiscuity and efficiency of this catalytic machinery so intriguing. For instance, Asp48 of EcCM is known to form a salt bridge to correctly position the catalytically important Arg11′ (Arg15′ in PchB; Figure 2).^23^ A salt bridge is not possible for the homologous Ala51 of WT PchB; yet, CM activity still proceeds, albeit at a much lower rate, suggesting that this interaction is neither imperative for CM nor for IPL activity in PchB. Positions 86 and 87 are clearly conserved in the CM-dominated AroQ class as Ala and Ser, respectively, but both 5-1 and 2-43 variants deviate from this consensus based on the 238 compared AroQ family members (Figure 3). Still, the selected Cys87 in these PchB variants is chemically similar to the consensus Ser and is therefore likely a CM-promoting replacement. The catalytic benefit of Ser87 versus Cys87 may indeed be just 1.3-fold in *k*_cat_/*K*_m_, as deduced from comparing variants 10-37 (RES) and 5-1 (REC). Interestingly, the Arg88 found in both clones preserves the positive charge of the lysine that represents the AroQ consensus residue (Figure 3). The substitution of the WT residue Ile at position 88 may lead to considerable conformational flexibility in a region proximal to the active site, as has been observed for the crystal structure of the Ile88Thr variant of PchB (I87T according to crystal structure numbering in PDB ID: 3HGW).^7^ Enhanced structural mobility may shift the conformational ensemble toward one that is less optimal for IPL but potentially more favorable for CM activity. This supports the idea of functional plasticity, where the enzyme can balance multiple activities through subtle structural shifts. At position 90, both 5-1 and 2-43 feature an alanine rather than the original glutamic acid of PchB. Although a hydrophobic residue is phylogenetically predominant at this position in the Pfam alignment (Figure 3), this is not essential as 17 of 19 different amino acid substitutions at position 90 were compatible with CM activity in a previous independent combinatorial mutagenesis and selection experiment with PchB.^3^

### Consequences for IPL Catalysis upon Evolving CM Activity

By selecting for efficient CM catalysis, the IPL activity of PchB library members dropped by 4–5 orders of magnitude. This is not surprising as there was no selective pressure to maintain the IPL function. On the contrary, a lowered IPL activity should even have benefitted clones in our selection scheme. In fact, in a previous growth selection experiment, a PchB variant with a spontaneous single Ile88Thr exchange had emerged for which IPL activity could no longer be detected whereas its weak CM catalysis was not affected.^2^ An independent study has confirmed a preserved CM and a 3-fold lower IPL *k*_cat_/*K*_m_ for this variant.^7^ As a result of the mutation, the selected mutant strain carrying the Ile88Thr PchB exhibited a doubled growth rate under Phe and Tyr-limiting conditions.^2^ It was speculated that this mutation favored growth in minimal medium because it prevented disadvantageous draining of the chorismate pool. The underlying rationale is that the introduction of a very efficient IPL into enabled a highly exergonic metabolic sequence from chorismate to the aromatic compound salicylate plus pyruvate, catalyzed by the combined action of the endogenous enzyme isochorismate synthase^30,31^ and the added IPL (Figure 1). Any mutation that impairs the IPL activity of the PchB introduced into the cell, such as the I88T exchange,^2,7^ will help preserving an elevated intracellular chorismate pool, which is in a well-balanced thermodynamic equilibrium with the isochorismate pool.^30,82^ The resulting higher cytoplasmic chorismate concentration may ultimately lead to conversion into sufficient Phe and Tyr by the sloppy CM activity inherent in wild-type PchB to allow for decent growth of the selection strain, which was otherwise lacking its endogenous CMs.^2^ Likewise, our particular directed evolution scheme should simultaneously have selected for increased CM and decreased IPL activity. This can explain a higher fitness, as reflected by better growth on minimal media, for several of the selected PchB library clones despite not encoding significantly improved CMs. The higher selection pressure applied here compared to the previous growth selection experiment,^2^ in combination with the more diverse libraries of enzyme variants probed in the laboratory evolution cycles, finally led to a different evolutionary trajectory. Under these conditions, we find that the evolution of higher CM activity, rather than loss of IPL activity in PchB, was the decisive advantage for survival.

## Conclusions

Of the natural AroQ class CMs screened in this work for promiscuous catalysis only *MtCM showed detectable IPL activity (Table 4). When scrutinizing active site residues that may be responsible for this, we found that valine (Val55 in PchB) is common to *MtCM^40^ and PchB, whereas all other tested natural CMs have a glutamate at this position.^23,33,52,73,74^ In the PchB evolution experiment described here, emergence of Glu55 instead of the wild-type valine correlated with an increase in CM activity ranging from 2 to 40-fold in the evolved variants. Replacement of the Val by the AroQ consensus Glu by site-directed mutagenesis resulted in a dramatic drop in the IPL activity of wild-type PchB and reduced the spurious IPL catalysis by *MtCM further. However, since the isolated single Val-to-Glu exchanges have also impaired the corresponding CM activities of PchB and *MtCM, we assume that they significantly compromised the active site structure in both cases in the absence of compensating amino acid changes. Such enabling additional mutations were readily accessible with our targeted evolutionary approach. Alternatively, novel protein sequences that are compatible with functionally competent CM active sites can also be obtained by applying sequence-based statistical models. In a recent report, aggregated AroQ sequence data was used to computationally extract the critically needed constraints inherent in homologous sequences to ultimately design diverse nature-like CM catalysts.^83^

In the alignment of native AroQ sequences, ca. 80% have a glutamate at the position corresponding to Val55 in PchB (Figure 3), but valine is with 8% the second most frequent residue. Like *MtCM, valine-containing AroQ proteins may be candidates for natural enzymes with (spurious) promiscuous IPL activity. Such enzymes could exemplify evolutionary steppingstones for transforming a CM, required in primary metabolism for making two of the 20 standard proteinogenic amino acids, into a specialized IPL to serve in secondary metabolism.^31^ Our experiments, involving targeted random mutagenesis based on simple structural comparisons of active site models, have demonstrated that modest secondary or moonlighting activities can be successfully enhanced within just two evolutionary cycles to levels rivaling dedicated catalysts. In addition, they have established the close evolutionary relationship between CM and IPL, which catalyze mechanistically distinct pericyclic reactions.

## Supplementary Material



## Notes

Kinetic plots proving the absence of contaminating chorismate in isochorismate preparations (Supplementary Figure S1); alignment of the six bona fide annotated IPL protein sequences (Supplementary Figure S2); phylogenetic tree of the bacterial species that contain the annotated IPL proteins (Supplementary Figure S3); Michaelis–Menten plots of kinetic assays for WT PchB and for PchB variants selected after Cycle 1 (Supplementary Figure S4); Michaelis–Menten plots of CM kinetic assays for PchB variants selected after Cycle 2 (Supplementary Figure S5); Michaelis–Menten plots of IPL kinetic assays for PchB variants selected after Cycle 2 (Supplementary Figure S6); experimental evidence for enzymatic IPL activity of *MtCM above background (Supplementary Figure S7); and Michaelis–Menten plot showing IPL activity of *MtCM for independent biological replicates (Supplementary Figure S8) (PDF)

## Notes

MjCM (UniProtKB ID: Q57696); *MtCM (UniProtKB ID: P9WIB9); *PaeCM (UniProtKB ID: Q9HU05); PchB (UniProtKB ID: Q51507); ScCM (UniProtKB ID: P32178).

## Abbreviations

CM: chorismate mutase
EcCM: chorismate mutase domain (AroQ_α_ subclass) of the bifunctional chorismate mutase-prephenate dehydratase
IPL: isochorismate pyruvate lyase
IPTG: isopropyl-1-thio-β-d-galactopyranoside
MjCM': proteolytically stable version of the chorismate mutase (AroQ_α_ subclass) from *Methanocaldococcus* (formerly called *Methanococcus*) *jannaschii*
mMjCM: engineered monomeric variant of the chorismate mutase from
*MtCM: secreted chorismate mutase (AroQ_γ_ subclass) from
*PaeCM: secreted chorismate mutase (AroQ_γ_ subclass) from
PchB: enzyme responsible for isochorismate pyruvate lyase activity
PCR: polymerase chain reaction
PDB: Protein Data Bank
rmsd: root-mean-square deviation
ScCM: chorismate mutase (AroQ_β_ subclass) from
WT: wild-type

## References

[1] Serino L., Reimmann C., Visca P., Beyeler M., Della Chiesa V., Haas D. (1997). Biosynthesis of pyochelin and dihydroaeruginoic acid requires the
iron-regulated *pchDCBA* operon in *Pseudomonas
aeruginosa*. J. Bacteriol..

[2] Gaille C., Kast P., Haas D. (2002). Salicylate
biosynthesis in *Pseudomonas aeruginosa*. Purification
and characterization
of PchB, a novel bifunctional enzyme displaying isochorismate pyruvate
lyase and chorismate mutase activities. J. Biol.
Chem..

[3] Künzler D. E., Sasso S., Gamper M., Hilvert D., Kast P. (2005). Mechanistic
insights into the isochorismate pyruvate lyase activity of the catalytically
promiscuous PchB from combinatorial mutagenesis and selection. J. Biol. Chem..

[4] DeClue M. S., Baldridge K. K., Kast P., Hilvert D. (2006). Experimental and computational
investigation of the uncatalyzed rearrangement and elimination reactions
of isochorismate. J. Am. Chem. Soc..

[5] DeClue M. S., Baldridge K. K., Künzler D. E., Kast P., Hilvert D. (2005). Isochorismate
pyruvate lyase: A pericyclic reaction mechanism?. J. Am. Chem. Soc..

[6] Zaitseva J., Lu J., Olechoski K. L., Lamb A. L. (2006). Two crystal structures of the isochorismate
pyruvate lyase from *Pseudomonas aeruginosa*. J. Biol. Chem..

[7] Luo Q., Olucha J., Lamb A. L. (2009). Structure-function analyses of isochorismate-pyruvate
lyase from *Pseudomonas aeruginosa* suggest differing
catalytic mechanisms for the two pericyclic reactions of this bifunctional
enzyme. Biochemistry.

[8] Olucha J., Ouellette A. N., Luo Q., Lamb A. L. (2011). pH Dependence of
catalysis by *Pseudomonas aeruginosa* isochorismate-pyruvate
lyase: Implications for transition state stabilization and the role
of lysine 42. Biochemistry.

[9] Luo Q., Meneely K. M., Lamb A. L. (2011). Entropic
and enthalpic components
of catalysis in the mutase and lyase activities of *Pseudomonas
aeruginosa* PchB. J. Am. Chem. Soc..

[10] Olucha J., Meneely K. M., Lamb A. L. (2012). Modification
of residue 42 of the
active site loop with a lysine-mimetic side chain rescues isochorismate-pyruvate
lyase activity in *Pseudomonas aeruginosa* PchB. Biochemistry.

[11] Martí S., Andrés J., Moliner V., Silla E., Tuñón I., Bertrán J. (2009). Mechanism and plasticity of isochorismate pyruvate
lyase: A computational study. J. Am. Chem. Soc..

[12] Lamb A. L. (2011). Pericyclic
reactions catalyzed by chorismate-utilizing enzymes. Biochemistry.

[13] Copley S. D., Knowles J. R. (1985). The uncatalyzed Claisen rearrangement of chorismate
to prephenate prefers a transition state of chairlike geometry. J. Am. Chem. Soc..

[14] Addadi L., Jaffe E. K., Knowles J. R. (1983). Secondary tritium isotope effects
as probes of the enzymic and nonenzymic conversion of chorismate to
prephenate. Biochemistry.

[15] Sogo S. G., Widlanski T. S., Hoare J. H., Grimshaw C. E., Berchtold G. A., Knowles J. R. (1984). Stereochemistry of the rearrangement of chorismate
to prephenate: Chorismate mutase involves a chair transition state. J. Am. Chem. Soc..

[16] Gajewski J. J., Jurayj J., Kimbrough D. R., Gande M. E., Ganem B., Carpenter B. K. (1987). On the mechanism of rearrangement of chorismic acid
and related compounds. J. Am. Chem. Soc..

[17] Kast P., Asif-Ullah M., Jiang N., Hilvert D. (1996). Exploring the active
site of chorismate mutase by combinatorial mutagenesis and selection:
The importance of electrostatic catalysis. Proc.
Natl. Acad. Sci. U.S.A..

[18] Gustin D. J., Mattei P., Kast P., Wiest O., Lee L., Cleland W. W., Hilvert D. (1999). Heavy atom isotope effects reveal
a highly polarized transition state for chorismate mutase. J. Am. Chem. Soc..

[19] Kienhöfer A., Kast P., Hilvert D. (2003). Selective stabilization of the chorismate
mutase transition state by a positively charged hydrogen bond donor. J. Am. Chem. Soc..

[20] Wright S. K., DeClue M. S., Mandal A., Lee L., Wiest O., Cleland W. W., Hilvert D. (2005). Isotope effects on the enzymatic
and nonenzymatic reactions of chorismate. J.
Am. Chem. Soc..

[21] Chook Y. M., Ke H., Lipscomb W. N. (1993). Crystal structures of the monofunctional chorismate
mutase from *Bacillus subtilis* and its complex with
a transition state analog. Proc. Natl. Acad.
Sci. U.S.A..

[22] Chook Y. M., Gray J. V., Ke H., Lipscomb W. N. (1994). The monofunctional
chorismate mutase from *Bacillus subtilis*: Structure
determination of chorismate mutase and its complexes with a transition
state analog and prephenate, and implications for the mechanism of
the enzymatic reaction. J. Mol. Biol..

[23] Lee A. Y., Karplus P. A., Ganem B., Clardy J. (1995). Atomic structure of
the buried catalytic pocket of *Escherichia coli* chorismate
mutase. J. Am. Chem. Soc..

[24] Sträter N., Schnappauf G., Braus G., Lipscomb W. N. (1997). Mechanisms of catalysis
and allosteric regulation of yeast chorismate mutase from crystal
structures. Structure.

[25] Ökvist M., Dey R., Sasso S., Grahn E., Kast P., Krengel U. (2006). 1.6 Å
Crystal structure of the secreted chorismate mutase from *Mycobacterium
tuberculosis*: Novel fold topology revealed. J. Mol. Biol..

[26] Kast P., Grisostomi C., Chen I. A., Li S., Krengel U., Xue Y., Hilvert D. (2000). A strategically positioned
cation is crucial for efficient
catalysis by chorismate mutase. J. Biol. Chem..

[27] Sasso S., Ökvist M., Roderer K., Gamper M., Codoni G., Krengel U., Kast P. (2009). Structure and function of a complex
between chorismate mutase and DAHP synthase: Efficiency boost for
the junior partner. EMBO J..

[28] Bentley R. (1990). The Shikimate
Pathway – A Metabolic Tree with Many Branches. Crit. Rev. Biochem. Mol. Biol..

[29] Haslam, E. Shikimic Acid: Metabolism and Metabolites; John Wiley & Sons, 1993.

[30] Shelton C.
L., Lamb A. L. (2018). Unraveling
the structure and mechanism of the MST­(ery)
enzymes. Trends Biochem. Sci..

[31] Hubrich F., Müller M., Andexer J. N. (2021). Chorismate- and isochorismate converting
enzymes: Versatile catalysts acting on an important metabolic node. Chem. Commun..

[32] Gu W., Williams D. S., Aldrich H. C., Xie G., Gabriel D. W., Jensen R. A. (1997). The AroQ and PheA domains of the bifunctional P-protein
from *Xanthomonas campestris* in a context of genomic
comparison. Microb. Comp. Genomics.

[33] MacBeath G., Kast P., Hilvert D. (1998). A small, thermostable,
and monofunctional
chorismate mutase from the archaeon *Methanococcus jannaschii*. Biochemistry.

[34] Roderer K., Kast P. (2009). Evolutionary cycles
for pericyclic reactions – Or why we keep
mutating mutases. Chimia.

[35] Kast P., Hilvert D. (1996). Genetic selection strategies
for generating and characterizing
catalysts. Pure Appl. Chem..

[36] Kast P., Hilvert D. (1997). 3D structural information
as a guide to protein engineering
using genetic selection. Curr. Opin. Struct.
Biol..

[37] Taylor S. V., Kast P., Hilvert D. (2001). Investigating and engineering enzymes
by genetic selection. Angew. Chem. Int. Ed.
Engl..

[38] Kast P., Asif-Ullah M., Hilvert D. (1996). Is chorismate mutase a prototypic
entropy trap? – Activation parameters for the *Bacillus
subtilis* enzyme. Tetrahedron Lett..

[39] MacBeath G., Kast P. (1998). UGA read-through artifacts
— When popular gene expression
systems need a pATCH. Biotechniques.

[40] Sasso S., Ramakrishnan C., Gamper M., Hilvert D., Kast P. (2005). Characterization
of the secreted chorismate mutase from the pathogen *Mycobacterium
tuberculosis*. FEBS J..

[41] Schmidt K., Leistner E. (1995). Microbial production
of (+)-*trans*-isochorismic
acid. Biotechnol. Bioeng..

[42] Balakrishnan R., Backman K. (1988). Controllable alteration
of cell genotype in bacterial
cultures using an excision vector. Gene.

[43] Miller, J. H. A Short Course in Bacterial Genetics. A Laboratory Manual and Handbook for Escherichia coli and Related Bacteria; Cold Spring Harbor Laboratory, 1992.

[44] Iida S., Arber W. (1980). On the role of IS*1* in the formation of hybrids between
the bacteriophage P1 and the R plasmid NR1. Mol. Gen. Genet..

[45] Roderer K., Neuenschwander M., Codoni G., Sasso S., Gamper M., Kast P. (2014). Functional mapping of protein-protein interactions in an enzyme complex
by directed evolution. PLoS One.

[46] Gamper M., Hilvert D., Kast P. (2000). Probing the
role of the C-terminus
of *Bacillus subtilis* chorismate mutase by a novel
random protein-termination strategy. Biochemistry.

[47] Mailand, S. The exported chorismate mutase from bacterial pathogens of mammals: From residues important for protein export to the quest for the biological function. Ph.D. Thesis, ETH Zurich, 2018.

[48] Sambrook, J. ; Russell, D. W. Molecular Cloning: A Laboratory Manual; Cold Spring Harbor Laboratory, 2001.

[49] Sikorski R.
S., Boeke J. D. (1991). In vitro
mutagenesis and plasmid shuffling: From cloned
gene to mutant yeast. Methods Enzymol..

[50] Sharp P. M., Cowe E., Higgins D. G., Shields D. C., Wolfe K. H., Wright F. (1988). Codon usage patterns
in *Escherichia coli*, *Bacillus subtilis*, *Saccharomyces cerevisiae*, *Schizosaccharomyces
pombe*, *Drosophila
melanogaster* and *Homo sapiens*; a review
of the considerable within-species diversity. Nucleic Acids Res..

[51] Patrick W. M., Firth A. E., Blackburn J. M. (2003). User-friendly algorithms for estimating
completeness and diversity in randomized protein-encoding libraries. Protein Eng..

[52] MacBeath G., Kast P., Hilvert D. (1998). Redesigning enzyme topology by directed
evolution. Science.

[53] Smith P. K., Krohn R. I., Hermanson G. T., Mallia A. K., Gartner F. H., Provenzano M. D., Fujimoto E. K., Goeke N. M., Olson B. J., Klenk D. C. (1985). Measurement
of protein using bicinchoninic acid. Anal. Biochem..

[54] Grisostomi C., Kast P., Pulido R., Huynh J., Hilvert D. (1997). Efficient
in vivo synthesis and rapid purification of chorismic acid using an
engineered *Escherichia coli* strain. Bioorg. Chem..

[55] Mattei P., Kast P., Hilvert D. (1999). *Bacillus subtilis* chorismate mutase is partially diffusion-controlled. Eur. J. Biochem..

[56] Helmstaedt K., Heinrich G., Lipscomb W. N., Braus G. H. (2002). Refined molecular
hinge between allosteric and catalytic domain determines allosteric
regulation and stability of fungal chorismate mutase. Proc. Natl. Acad. Sci. U.S.A..

[57] Bateman A., Coin L., Durbin R., Finn R. D., Hollich V., Griffiths-Jones S., Khanna A., Marshall M., Moxon S., Sonnhammer E. L. L. (2004). The Pfam protein families database. Nucleic Acids Res..

[58] Abramson J., Adler J., Dunger J., Evans R., Green T., Pritzel A., Ronneberger O., Willmore L., Ballard A. J., Bambrick J. (2024). Accurate
structure prediction of biomolecular
interactions with AlphaFold 3. Nature.

[59] Iwaki T., Kawamura A., Ishino Y., Kohno K., Kano Y., Goshima N., Yara M., Furusawa M., Doi H., Imamoto F. (1996). Preferential replication-dependent
mutagenesis in the
lagging DNA strand in *Escherichia coli*. Mol. Gen. Genet..

[60] Segel, I. H. Biochemical Calculations: How to Solve Mathematical Problems in General Biochemistry; John Wiley & Sons, 1976.

[61] Stocker C., Khatanbaatar T., Bressan L., Würth-Roderer K., Cordara G., Krengel U., Kast P. (2023). Novel exported fusion
enzymes with chorismate mutase and cyclohexadienyl dehydratase activity:
Shikimate pathway enzymes teamed up in no man’s land. J. Biol. Chem..

[62] Ohno S., Wolf U., Atkin N. B. (1968). Evolution from fish
to mammals by
gene duplication. Hereditas.

[63] Goodman M., Moore G. W., Matsuda G. (1975). Darwinian
evolution in the genealogy
of haemoglobin. Nature.

[64] Hughes A. L. (1994). The evolution
of functionally novel proteins after gene duplication. Proc. R. Soc. Lond. B.

[65] Piatigorsky J., Wistow G. (1991). The recruitment of
crystallins: New functions precede
gene duplication. Science.

[66] Taylor J. S., Raes J. (2004). Duplication and divergence:
The evolution of new genes and old ideas. Annu.
Rev. Genet..

[67] Aharoni A., Gaidukov L., Khersonsky O., McQ Gould S., Roodveldt C., Tawfik D. S. (2005). The ‘evolvability’
of promiscuous protein functions. Nat. Genet..

[68] Copley S. D. (2020). Evolution
of new enzymes by gene duplication and divergence. FEBS J..

[69] Copley S. D. (2003). Enzymes
with extra talents: Moonlighting functions and catalytic promiscuity. Curr. Opin. Chem. Biol..

[70] Gupta M. N., Uversky V. N. (2023). Moonlighting enzymes: When cellular
context defines
specificity. Cell. Mol. Life Sci..

[71] Singh N., Bhalla N. (2020). Moonlighting proteins. Annu.
Rev. Genet..

[72] Werelusz P., Galiniak S., Mołoń M. (2024). Molecular functions of moonlighting
proteins in cell metabolic processes. Biochim.
Biophys. Acta. Mol. Cell Res..

[73] Xue Y., Lipscomb W. N. (1995). Location of the active site of allosteric chorismate
mutase from *Saccharomyces cerevisiae*, and comments
on the catalytic and regulatory mechanisms. Proc. Natl. Acad. Sci. U.S.A..

[74] Xue Y., Lipscomb W. N., Graf R., Schnappauf G., Braus G. (1994). The crystal structure of allosteric chorismate mutase at 2.2-Å
resolution. Proc. Natl. Acad. Sci. U.S.A..

[75] Burschowsky D., Thorbjo̷rnsrud H. V., Heim J. B., Fahrig-Kamarauskaitė J., Würth-Roderer K., Kast P., Krengel U. (2018). Inter-enzyme allosteric
regulation of chorismate mutase in *Corynebacterium glutamicum*: Structural basis of feedback activation by Trp. Biochemistry.

[76] Kast P., Hartgerink J. D., Asif-Ullah M., Hilvert D. (1996). Electrostatic catalysis
of the Claisen rearrangement: Probing the role of Glu78 in *Bacillus subtilis* chorismate mutase by genetic selection. J. Am. Chem. Soc..

[77] Cload S. T., Liu D. R., Pastor R. M., Schultz P. G. (1996). Mutagenesis study
of active site residues in chorismate mutase from *Bacillus
subtilis*. J. Am. Chem. Soc..

[78] Liu D. R., Cload S. T., Pastor R. M., Schultz P. G. (1996). Analysis of active
site residues in *Escherichia coli* chorismate mutase
by site-directed mutagenesis. J. Am. Chem. Soc..

[79] Kaltenbach M., Jackson C. J., Campbell E. C., Hollfelder F., Tokuriki N. (2015). Reverse evolution leads to genotypic
incompatibility
despite functional and active site convergence. Elife.

[80] Starr T. N., Thornton J. W. (2016). Epistasis in protein evolution. Prot. Sci..

[81] Johnson M. S., Reddy G., Desai M. M. (2023). Epistasis and evolution: Recent advances
and an outlook for prediction. BMC Biol..

[82] Tewari Y. B., Davis A. M., Reddy P. T., Goldberg R. N. (2000). A thermodynamic
study of the conversion of chorismate to isochorismate. J. Chem. Thermodyn..

[83] Russ W. P., Figliuzzi M., Stocker C., Barrat-Charlaix P., Socolich M., Kast P., Hilvert D., Monasson R., Cocco S., Weigt M. (2020). An evolution-based model
for designing chorismate mutase enzymes. Science.

