# Supplementary material for: (Reverse) Evolution of a Promiscuous Isochorismate Pyruvate Lyase into an Efficient Chorismate Mutase
Source: Biochemistry. 2025 Jul 22;64(15):3459–73. doi: 10.1021/acs.biochem.5c00157 (PMC12329711; doi:10.1021/acs.biochem.5c00157)
Supplement: Supplementary file 1 [file bi5c00157_si_001.pdf]

Supporting Information

(Reverse) Evolution of a Promiscuous Isochorismate Pyruvate Lyase into an Efficient Chorismate Mutase

Dominik E. Künzler,[§] Luca Bressan,[§] Linda Jäger,[§] Marianne Gamper, and Peter Kast*

Laboratory of Organic Chemistry, ETH Zurich, CH-8093 Zurich, Switzerland

[§] D.E.K., L.B., and L.J. contributed equally to this work.

* To whom correspondence should be addressed. Laboratory of Organic Chemistry, ETH Zurich, HCI F 333, Vladimir-Prelog-Weg 3, CH-8093 Zurich, Switzerland.
E-mail: kast@org.chem.ethz.ch. Phone: +41-44-632-2908.

Supplementary Figures

Figure S1

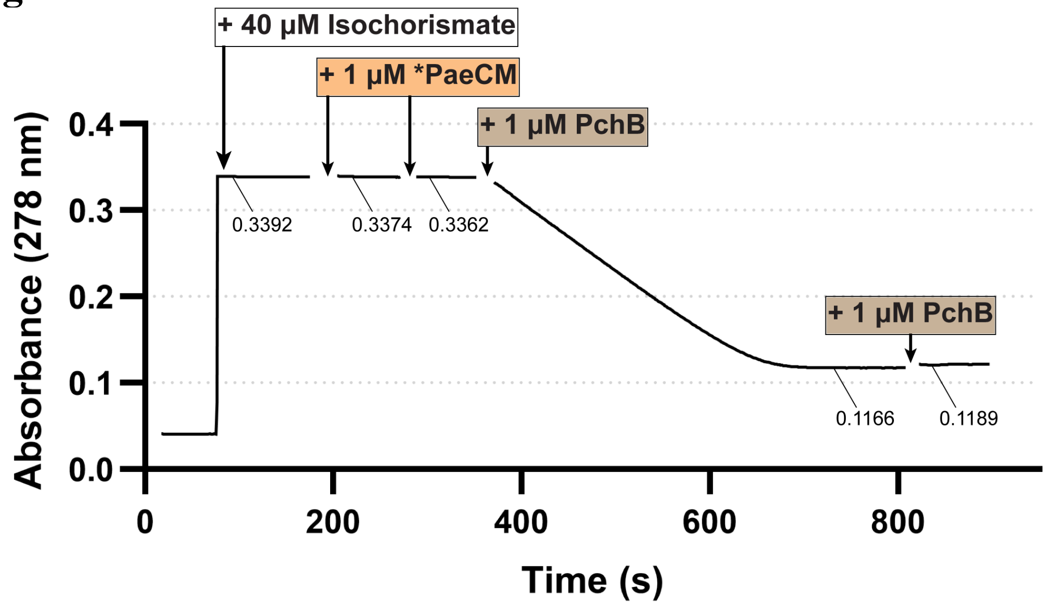

Supplementary Figure S1. Isochorismate depletion assay with *PaeCM and PchB enzymes. The absorbance at 278 nm of a 40 μM isochorismate solution in 50 mM potassium phosphate buffer, pH 7.5, was monitored at 30 °C. Two subsequent additions of 1 μM *PaeCM were followed by two additions of 1 μM PchB. Selected absorbance values recorded at different stages of the assay are indicated below the experimental curve. The indicated concentrations refer to the final concentrations in the quartz cuvette.

Figure S2

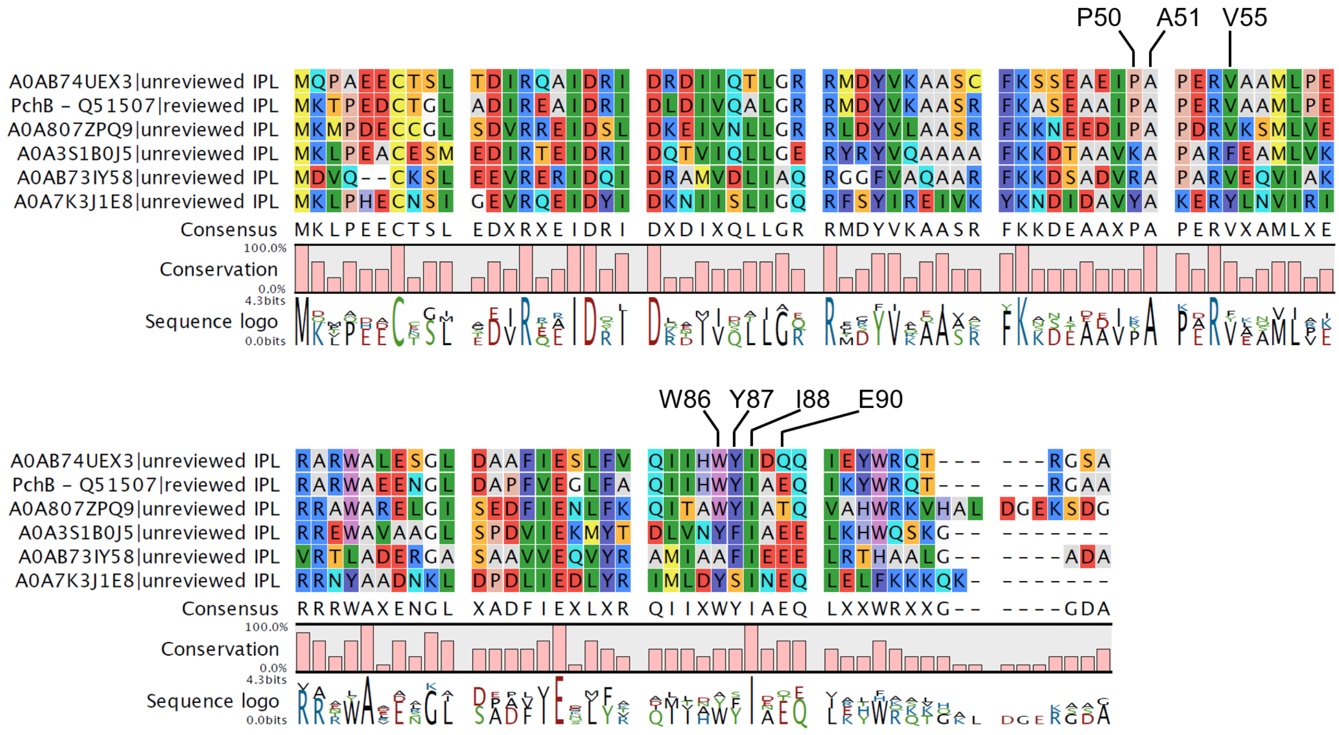

Supplementary Figure S2. Aligment of the six bona fide IPL protein sequences that were retrieved from the preassembled Pfam database when searching the AroQ domain compilation with the key words "isochorismate" and "lyase". The entire AroQ domain assembly, named "Chorismate Mutase Type II domain family" (https://www.ebi.ac.uk/interpro/entry/pfam/PF01817/), consists of at least 53,000 homologous proteins. Whereas annotated IPL are extremely rare among the AroQ-homologous entries, searching with "chorismate" and "mutase" yields at least 49,000 hits. Among the six aligned IPL-annotated sequences is PchB studied here, which is the only biochemically established IPL adopting the AroQ-fold (second entry in the alignment, accession number Q51507). The other 5 proteins may or may not have IPL activity. The six sequences were aligned using Qiagen CLC Genomics Workbench Version 23.0.3 (https://digitalinsights.qiagen.com/) based on the fasta input file downloaded from the Pfam database. The randomized WT PchB residues are indicated above the aligned sequences, using the numbering defined in the main manuscript. The phylogenetic relationship of the 6 bona fide IPL sequences is shown in Supplementary Figure S3.

Figure S3

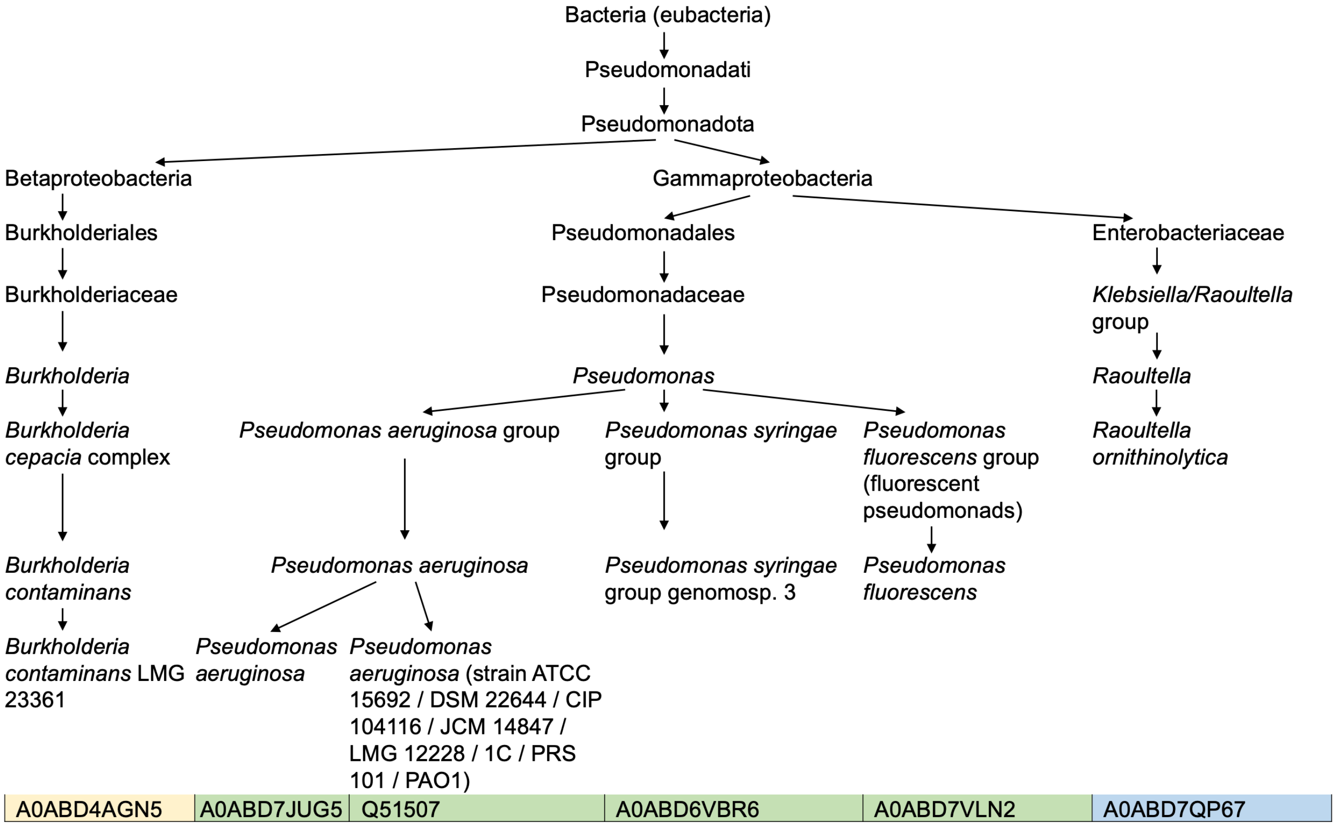

Supplementary Figure S3. Phylogenetic context of the source organisms of the six AroQ fold family protein sequences that were annotated as bona fide potential IPLs in the preassembled Pfam database (https://www.ebi.ac.uk/interpro/entry/pfam/PF01817/protein/UniProt/?search=isochorismate%20lyase#table). For details of the sequence retrieval, see Supplementary Figure S2. The taxonomic information for the species was taken for each individual sequence accession number (shown here in the last row and in Supplementary Figure S2) from https://www.ebi.ac.uk/interpro/search/text/. As can be seen in the phylogenetic tree, all annotated IPL sequences were found in bacterial species from the phylum Pseudomonadota, with the four annotated IPLs highlighted in olive green, including the biochemically established enzyme PchB (accession number Q51507), belonging to the genus *Pseudomonas*. In contrast, biochemically verified AroQ class CMs are ubiqitous among bacteria, archaea, fungi, and plants.

Figure S4A

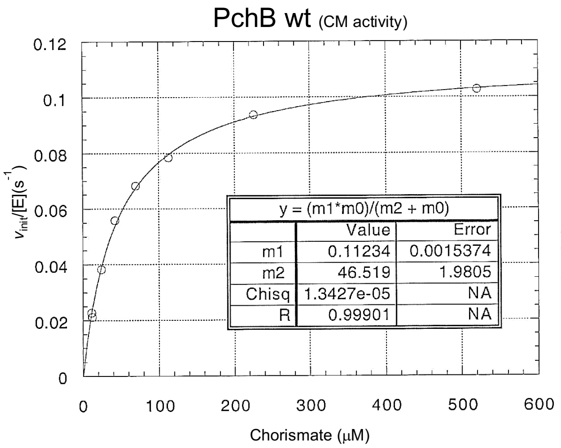

Figure S4B

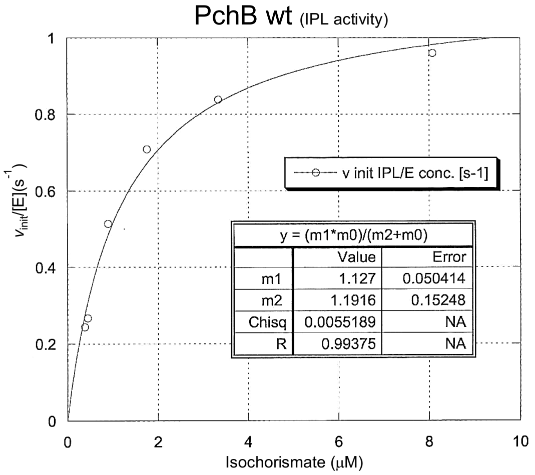

Figure S4C

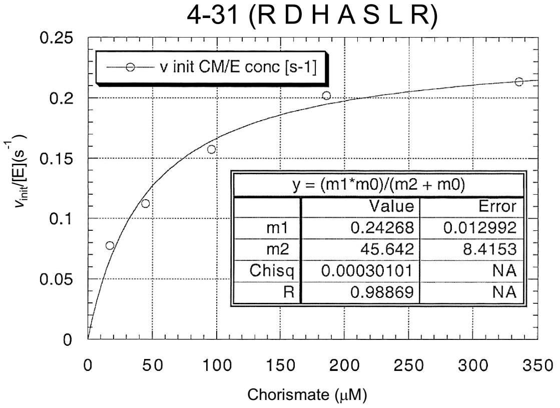

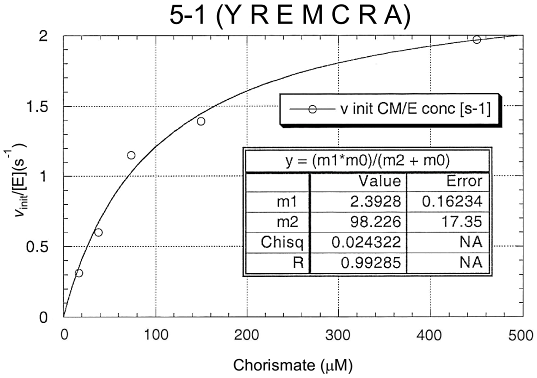

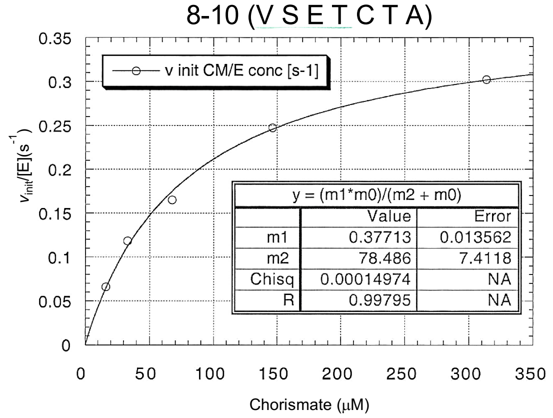

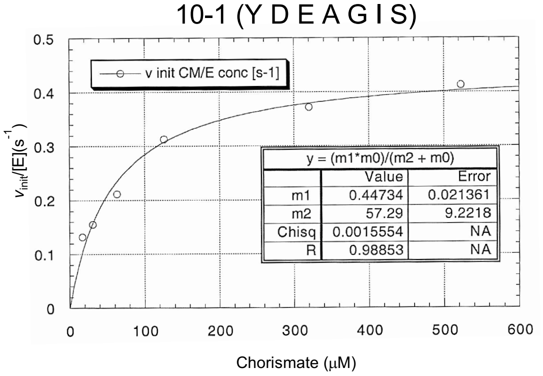

Supplementary Figure S4. Michaelis-Menten plots of kinetic assays for WT PchB and for PchB variants selected after Cycle 1. (A) CM activity of WT PchB. (B) IPL activity of WT PchB. (C) CM activity of PchB variants 4-31, 5-1, 8-10, and 10-1 that emerged from the evolutionary Cycle 1. Residues (one-letter code) selected upon randomizing WT PchB positions P50, A51, V55, W86, Y87, I88, and E90 are indicated in this sequence above the plots. The box shows m1 = k_{cat} (s^{-1}), m2 = K_m (μM), the plot's correlation coefficient R, and the error of the fit to the Michelis-Menten equation. For more details to the CM and IPL assays, see the legends to Supplementary Figures S5 and S6, respectively.

Figure S5

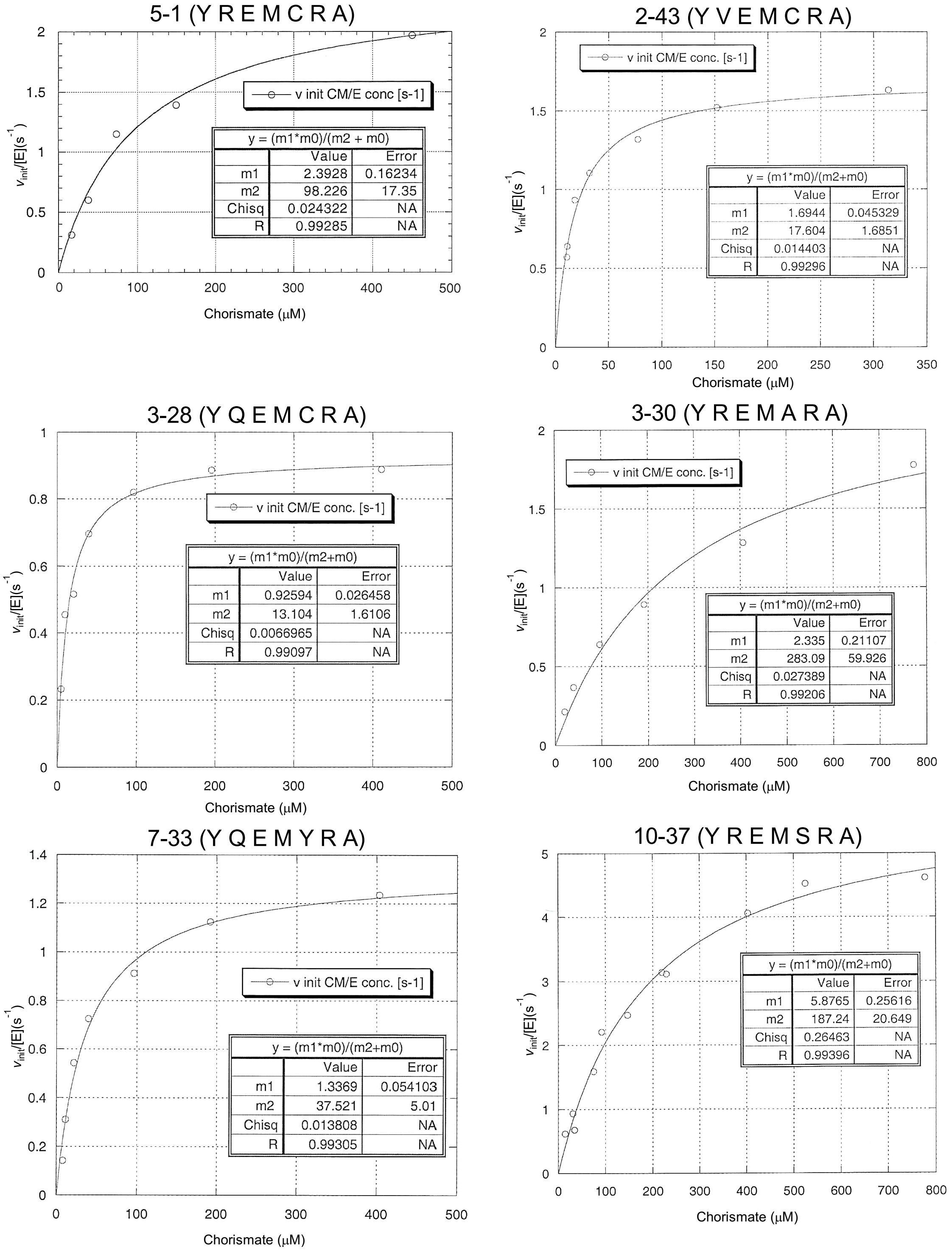

Supplementary Figure S5. Michaelis-Menten plots of CM kinetic assays for PchB variants selected after Cycle 2 and the parental variant 5-1 from Cycle 1 (top left). The initial velocity (v_{init}) was calculated for each data point from the consumption of chorismate monitored at $A_{274\text{ nm}}$ in 50 mM potassium phosphate buffer, pH 7.5, at 30°C by subtracting the uncatalyzed reaction. The v_{init} divided by the enzyme concentration [E] data were fitted to the Michaelis-Menten equation. The catalytic parameters, errors of the fit, and sequence features are displayed as indicated in the legend to Supplementary Figure S4.

Figure S6

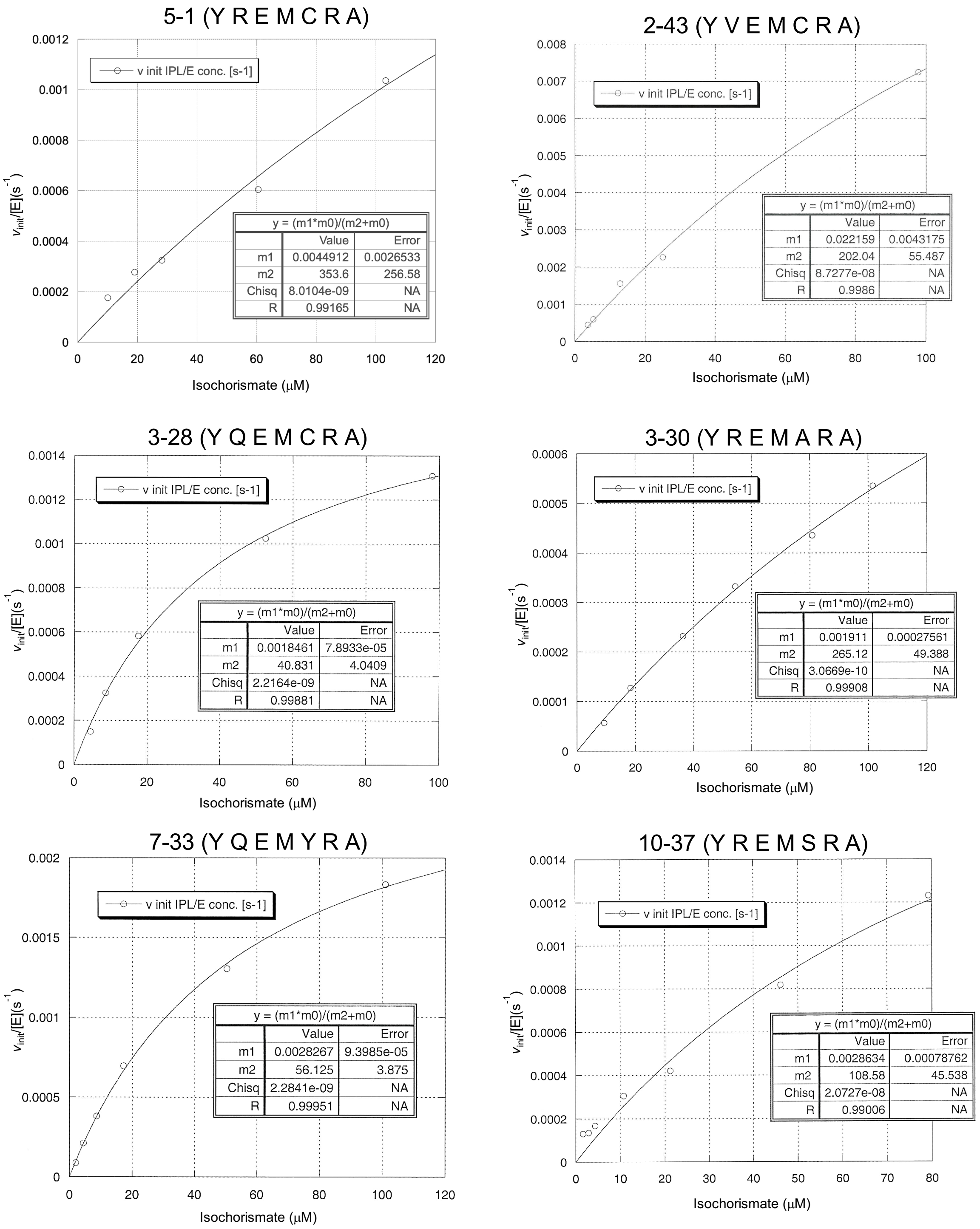

Supplementary Figure S6. Michaelis-Menten plots of IPL kinetic assays for PchB variants selected after Cycle 2 and the parental variant 5-1 from Cycle 1 (top left). For each data point, the initial velocity (v_{init}) was derived from the background reaction-corrected decrease in $A_{278\ nm}$ over time of the added isochorismate in 50 mM potassium phosphate buffer, pH 7.5, at 30°C. Division by the PchB concentration [E] and fitting to the Michaelis-Menten equation resulted in k_{cat} (m1, in s^{-1}) and K_m (m2, in μM). Errors of the fit and sequence features are displayed as indicated in the legend to Supplementary Figure S4.

Figure S7A

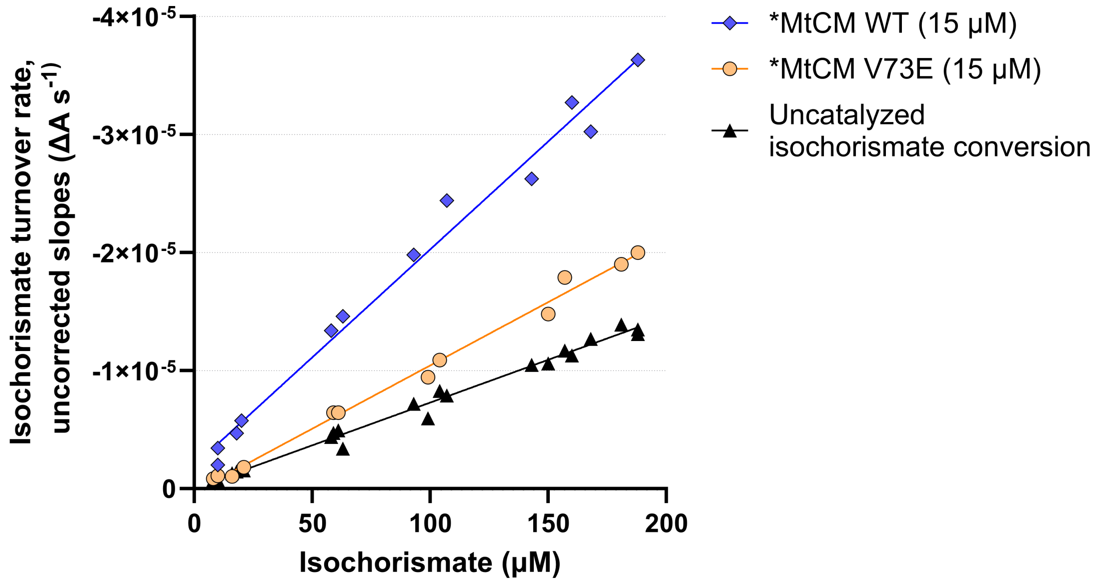

Figure S7B

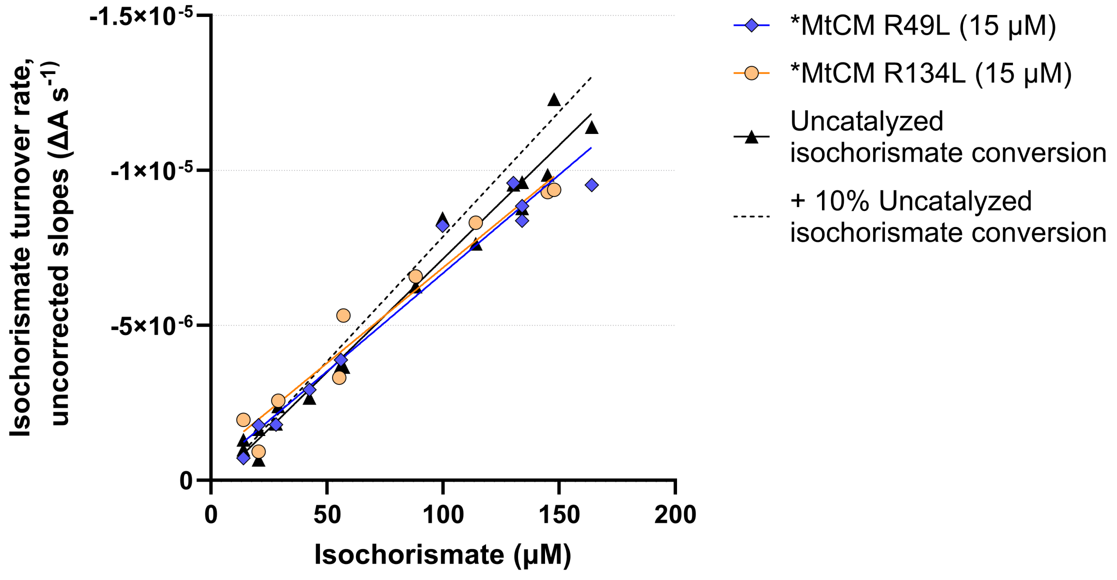

Supplementary Figure S7. IPL reaction observed with different *MtCM variants in comparison to the uncatalyzed substrate decay rate. Shown is the initial substrate consumption rate (without background correction), monitored at $A_{278\ nm}$ in 50 mM potassium phosphate, pH 7.5, at 30 °C for different isochorismate concentrations in the presence of the indicated enzyme variants and the corresponding uncatalyzed thermal decay in the absence of enzymes (black triangles). The solid lines represent a linear regression fit of the corresponding data points. (A) IPL reaction measured with WT *MtCM (blue diamonds) and with variant *MtCM V73E (orange circles) at the maximally feasible enzyme concentration (15 µM). (B) IPL reaction in presence of the active site-knockout variants *MtCM R49L (blue diamonds) and *MtCM R134L (orange circles). The dashed line simulates an activity 10% over the background for comparison.

Figure S8

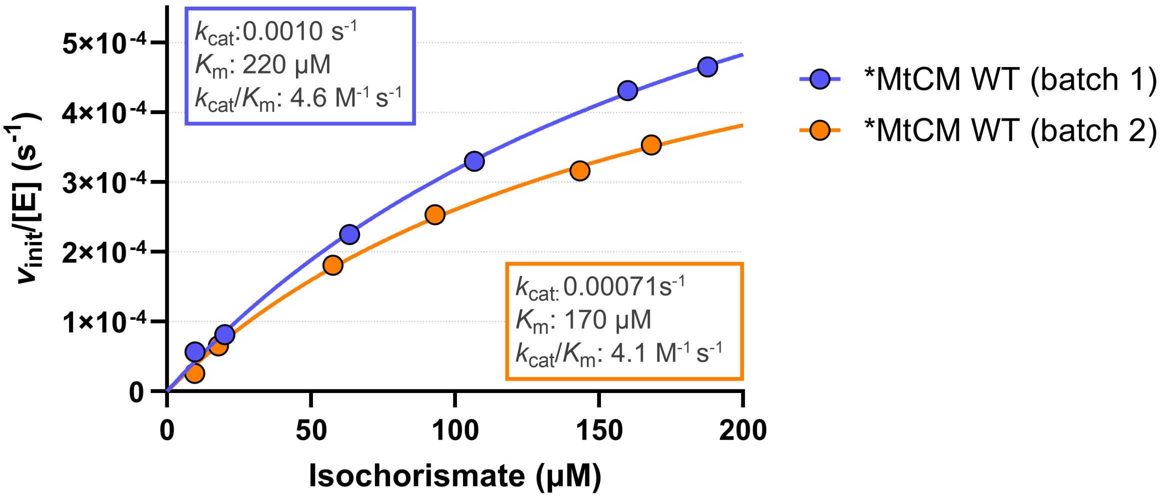

Supplementary Figure S8. IPL activity of biological replicates of WT *MtCM showing Michelis-Menten-type kinetics. Two independently prepared batches of Ni-NTA-purified WT *MtCM (blue or orange circles) were assayed for isochorismate turnover at 30 °C at different substrate concentrations. The curves represent the non-linear regression fit to the Michaelis-Menten equation for each set of data points. The boxes with the blue or orange frames show the respective calculated kinetic parameters. The average of the enzyme concentration-independent K_m values estimated for each batch is 200 ± 30 µM. The somewhat larger relative standard deviation for the averaged k_{cat} (0.00086 ± 0.00021 s^{-1}) parameter can be ascribed to varying impurities in the independent protein preparations combined with the typical errors when determining enzyme concentrations ([E]). The initial velocity (v_{init}) was calculated from the decrease in $A_{278\ nm}$ of the added isochorismate over time in 50 mM potassium phosphate buffer, pH 7.5, by subtracting the uncatalyzed thermal background reaction at the corresponding substrate concentration. More details are given in the Experimental Procedures section.
